# Need Fulfillment During Intergroup Contact: Three Experience Sampling Studies

**DOI:** 10.1177/01461672231204063

**Published:** 2023-11-21

**Authors:** Jannis Kreienkamp, Maximilian Agostini, Laura F. Bringmann, Peter de Jonge, Kai Epstude

**Affiliations:** 1 University of Groningen, The Netherlands; 2 Center for Peace and Conflict Studies, Wayne State University, USA

**Keywords:** intergroup contact, need fulfillment, outgroup attitudes, interaction quality, intensive longitudinal data

## Abstract

One challenge of modern intergroup contact research has been the question of when and why an interaction is perceived as positive and improves intergroup relations. We propose to consider the perceived fulfillment of the situationally most relevant need. We conducted three intensive longitudinal studies with recent migrants to capture their interactions with the majority out-group (*N_measurements_* = 10,297; *N_participants_* = 207). The situational need fulfillment mechanism is consistently a strong predictor of perceived interaction quality and positive out-group attitudes following intergroup interactions. The model is specific to out-group contact, robust to various need types, and works at least as well as Allport’s contact conditions. As one of the first studies to test intergroup contact theory using intensive longitudinal data, we offer insight into the mechanisms of positive intergroup contact during real-life interactions and find situational motivations to be a key building block for understanding and addressing positive intergroup interactions.

**Public significance statement**: In this article, we provide evidence that the fulfillment of situational needs during real-life intergroup contacts meaningfully predicts perceived interaction quality and positive outgroup attitudes. Methodologically, this offers a testament to the emerging practice of capturing real-life interactions using intensive longitudinal data. Theoretically, our results give weight to motivational fulfillment as a flexible and effective mechanism for understanding positive intergroup contact.

One of the main intergroup societal issues to date is the struggles of many migrants across the world, hoping to build a new life that includes a positive relationship with the majority group. The intergroup contact hypothesis postulates that prejudice can be reduced and favorable attitudes be increased if members of two groups have frequent and positive contact (e.g., Allport, 1954; Hewstone, 1996; Pettigrew, 1998). Over the past 70 years, a plethora of studies and interventions have shown the general effectiveness of positive intergroup contact (e.g., Pettigrew & Tropp, 2006). However, although a central assumption of intergroup contact theory is that contact should be positive, relatively little research has thus far explained when and why people perceive their everyday intergroup interactions as positive.

Importantly, as we still fail to understand when and why an interaction is perceived as positive, substantial theoretical and practical challenges remain. There is now consistent evidence that negative intergroup contacts lead to worse attitudes, prejudice, and reduced future interaction motivation (e.g., Barlow et al., 2012; Graf et al., 2014; Prati et al., 2021). In light of these findings, understanding interaction quality thus sits at the heart of understanding when an intergroup contact is successful (e.g., Allport, 1954; Brown et al., 2007; Tropp et al., 2016). But also in applied settings, policymakers and practitioners are thus far often under-prepared to deal with the occurrences of negative interactions, especially in everyday life contexts. Understanding the psychological mechanisms of when and why interactions are perceived as positive is, thus, an important issue for understanding whether an interaction leads to better intergroup perceptions, especially during everyday interactions.

We propose that one key to understanding how an interaction is perceived is to examine the level of need fulfillment it provides to an individual. As an example, if someone seeks acceptance by their interaction partner, and this need is fulfilled during the interaction, the person should rate the interaction and the group of the interaction partner more favorably. To test this idea, we collected three sets of real-life data from recent immigrants, assessing their daily interactions with majority group members, tracking situational needs, interaction quality, and outgroup attitudes.

## Need Mechanism in Intergroup Contact

Looking at the past literature, we can essentially separate intergroup contact theory research into a two-step problem. First, we need to understand when and why contact becomes a positive contact (contact *→* positive contact) and, second, we need to understand when and why positive contacts drive better intergroup relations (positive contact *→* better relations; e.g., see Allport, 1954; Hewstone, 1996; Pettigrew, 1998).

In recent years, research has focused on the second step of understanding the psychological processes that explain how positive contacts improve intergroup relations (e.g., see, Paolini et al., 2021). Among others, researchers have explored different forms of social categorizations (Pettigrew, 1998), the salience of social categories (Brown & Hewstone, 2005), intimacy (e.g., Marinucci et al., 2021) and attachment (e.g., Tropp, 2021), threat and intergroup anxiety (e.g., Stephan et al., 2008), and knowledge about the other group (Pettigrew & Tropp, 2008). Most recently, researchers have even looked at how empowerment needs fulfillment during positive intergroup contact can explain some of the beneficial intergroup effects (Hässler et al., 2021). There is thus, substantial evidence on the psychological mechanisms that explain the effects of positive contact.

Research on the first step of what makes an interaction positive, to begin with, tends to be much older and often more static and contextual. The most widely used approach has been the idea that equal status, common goals, collaboration, and structural support during the interaction form Allport’s optimal conditions for positive contacts (Allport, 1954; Pettigrew, 1969). Following Allport’s original conditions, several additional conditions of optimal contact were proposed, including, stereotype disconfirmation (Cook, 1978) or common language and voluntary interaction (Wagner & Machleit, 1986; for a critical discussion see Pettigrew, 1986). However, despite their prominence in guiding research on this topic, meeting the contact conditions does not seem to be necessary for finding positive effects of intergroup contact (Pettigrew & Tropp, 2006), and more fundamentally, the conditions often do not capture any underlying psychological mechanisms of why an interaction is perceived as positive (e.g., Pettigrew, 1998).

In this article, we focus on the role of motivation and need fulfillment to understand when and why exactly an interaction is perceived as positive. We propose need fulfillment in particular because needs are a fundamental aspect of the human experience that governs a significant number of emotional, cognitive, and behavioral facets (Kreienkamp et al., 2023; Kruglanski et al., 2002). Importantly, need fulfillment has particularly been highlighted in explaining the success of (close) relationships, psychosocial functioning, as well as reducing conflict between groups—all of which are essential to positive intergroup interactions.

On an individual psychological level, there is a long tradition of using need fulfillment to explain what drives human adaptation and social relations. From the early works of Maslow (1943) and Lewin (1926) to more recent works by Ryan and Deci (2017) or Steverink and Lindenberg (2006), the fulfillment of needs has been considered a driver of psychosocial functioning. Most relevant to our proposal here, within experience sampling studies need fulfillment has been found to explain variations in well-being during daily interactions (Downie et al., 2008) and has been found to be important in understanding the success of close relationships (e.g., see Knee & Browne, 2023). In short, an extensive body of scholarly work underscores the significance of need satisfaction in fostering favorable social relationships and social functioning.

Beyond the individual relations literature, need fulfillment has recently also seen application as a psychological mechanism in the intergroup relations literature. Social identity theory has focused on the role of self-esteem needs in understanding how people navigate intergroup contexts (e.g., Abrams & Hogg, 1988). In the study of conflict and reconciliation, addressing the differential needs of victims and perpetrators (i.e., the need for power and the need for morality, respectively) increased willingness to reconcile (Shnabel & Nadler, 2008). And similarly, addressing a relevant need for identity continuity among refugees in Turkey bolstered resilience in the face of discrimination experiences (Çelebi et al., 2017). In short, an increasing amount of literature is emphasizing the significance of need satisfaction in understanding intergroup dynamics.

It is thus not surprising that Dovidio and colleagues propose that: “To achieve truly constructive intergroup relations, it is important that intergroup exchanges meet the psychological needs of both majority- and minority-group members.” (Dovidio et al., 2017, p. 6). A call that has thus far remained unanswered when it comes to the basic tenet that needs fulfillment underpins positive and constructive interactions.

One reason why motivational considerations might have remained absent from the intergroup contact literature is that there is an overwhelming number of individual motives or goals that might be relevant to a person during an intergroup interaction. Researchers considering the motivational content would, thus, either test a few hyper-specific needs that might not be transferable to other intergroup contexts or they may need to assess a broad and diverse range of motives. However, while the specific need content differed within the different lines of research, what unites most motivational researchers is a focus on fulfilling the situationally relevant needs of people. This motivational experience of need fulfillment, thus, brings many of the diverse need content theories together and offers a common psychological mechanism for understanding positive intergroup contact.

Here it is important to briefly define what exactly we mean by need fulfillment and how it differs from need content theories. With motivation and need fulfillment we specifically mean the psychological experience of addressing an active and relevant need during the interaction. For our purposes, we define a need as:

**Definition 1 (Need):** A tension or deficiency in the organism that elicits a (non-specific) motivational force organizing affect, cognition, and behavior to reduce this unsatisfactory situation, which is to some extent necessary for the individual’s overall well-being—based on Dweck (2017), Hull (1943), Kruglanski et al. (2002), Lewin (1938), McClelland (1987), Ryan and Deci (2017), and Steverink and Lindenberg (2006).

The psychological experience of need fulfillment is, thus, distinct from the content of the need (i.e., the motive or goal). The content could, for example, include physical motives (such as safety or hygiene) but also psychosocial motives (such as acceptance or competence; e.g., see Pittman & Zeigler, 2007). The experience of needing is a more general process that arises when any important motive is thwarted or situationally active and relevant (Gollwitzer & Wicklund, 1985; Leander et al., 2020; Lewin, 1926). It is this perceived needing and the perceived fulfillment of needs that we focus on in this article. This is not to say that considering specific motives is irrelevant to contact situations but instead, we propose that the psychological experience of perceived need fulfillment is a core mechanism in understanding interaction quality perceptions, well-being, and out-group attitudes.

To test such a proposal, we can rely on adaptive and responsive survey designs that allow a tailored approach based on the participants’ inputs (e.g., Tourangeau et al., 2017). In particular, we propose to ask the participants to report their main goal during the interaction in a short open-ended question (i.e., name the situationally relevant need content), and with reference to their own response, the participants can then indicate how much this need was fulfilled during the interaction (i.e., need fulfillment mechanism). Such an adaptive approach allows us to take the initial step of testing whether situational need fulfillment indeed generally predicts perceived interaction quality, well-being, and positive outgroup attitudes independent of need content.

## Intergroup Contact in Daily Life

While we have argued that a need fulfillment mechanism is relevant to intergroup contact generally, its flexible and broad applicability might be ideally suited to address the pressing issue of understanding natural intergroup contacts outside the lab. Investigations of such “real-life interactions” often suffer from the difficulty that past intergroup contact research has either focused on the mechanisms of individual interactions in artificial lab studies (sometimes referred to as the intergroup interaction literature) or has focused on longer-term recall self-reports of natural interactions (commonly referred to as the intergroup contact literature; also see Pettigrew & Tropp, 2006). MacInnis and Page-Gould (2015) have even pointed out that these two approaches tend to find conflicting effects—where individual interactions (in the lab) have more negative effects and recall of real-world contact patterns have more positive effects for intergroup relations. We, thus, miss data following people in their diverse daily interactions and investigating the psychological mechanisms of contacts, especially as they compound over time. Even with extended intervention studies, the most fine-grained data available is usually limited to pre-post-control designs. It should be noted that there is an emerging body of literature looking at longitudinal effects with panel studies (e.g., Bracegirdle et al., 2023; Górska & Tausch, 2023). However, such studies still ask participants to recall their interactions of weeks, months, or years. The lack of longitudinal real-world data, however, stands in stark contrast to many of the theoretical advances that have focused on the dynamic nature of intergroup relations (e.g., Pettigrew, 1998), as well as the original contact hypothesis, which was focused on the daily interactions of people (Allport, 1954). As a result, prominent researchers in the field have long called for longitudinal (Pettigrew, 1998, 2008; Pettigrew & Tropp, 2011) and real-life experience-sampling data outside the lab (ESM MacInnis & Page-Gould, 2015; McKeown & Dixon, 2017). Such data would be able to capture real-life interactions that include interaction-specific mechanism information close to the actual experience.^1^

In the past, such data collections were often unfeasible because they were either physically impractical or too expensive. However, recent technological developments allow us to easily collect experience sampling data on mobile devices (e.g., Keil et al., 2020) or using web-based applications (e.g., Arslan et al., 2020). At the same time, analytical methods for such more complex data have become more readily available, making the analyses more approachable (e.g., see O’Donnell et al., 2021). Given these technological and methodological developments, we were able to collect three independent studies of extensive real-life data following the daily intergroup interactions of recently arrived migrants with the majority-group members.

## The Present Research

Using three independent sets of intensive longitudinal data (Studies 1–3), the aim of this article is essentially threefold. We (a) seek to test the basic ideas of the contact theory within real-world experience sampling data. We (b) aim to test the situational need fulfillment mechanism within the real-world data. And we (c) seek to ensure the stability, robustness, and embeddedness of our results.

First, for the general contact hypothesis test, our study is among the first to test the fundamental tenets of intergroup contact and Allport’s conditions in real-life intensive longitudinal data. Translating the contact hypothesis into intensive longitudinal data is not a trivial task, as past research traditions have used two fundamentally different approaches. While lab studies have tended to focus on the effect of a single positive interaction, cross-section studies have primarily investigated the frequency of positive interactions more generally. Intensive longitudinal data allows us to investigate both. We can test whether having a specific type of interaction vs. not having an interaction improves intergroup relations, but we can also use the participant’s 30-day contact reports to test whether participants with more positive interactions tend to benefit more from intergroup contact. Testing both approaches to the contact hypothesis allows us to go beyond replication of the basic theory but could disentangle individuals from aggregated contact effects and would allow for a direct comparison with both bodies of literature.

We test the basic contact hypothesis within and across the three studies. In particular, we assess the effect of individual interactions within each study using a multilevel model, but to avoid power limitations, we test the collective effect of contact frequency and quality after the individual studies, across all participants.

**Hypothesis 1:** Based on the most general understanding of the contact hypothesis, an increase in frequency and quality of contact should jointly account for more favorable outgroup attitudes within and across intensive longitudinal data.

The test of Allport’s conditions is notably restricted to measurements that report on out-group interactions because Allport’s conditions and interaction quality ratings cannot meaningfully be measured or imputed if participants did not have an interaction. Focusing on the interactions in detail, we use a multilevel regression model to test whether interactions that are higher in the fulfillment of Allport’s conditions predict more favorable outgroup attitudes. We would also expect that such interactions are perceived as higher in interaction quality.

**Hypothesis 2:** Based on the literature about Allport’s optimal contact conditions, intergroup interactions that are higher in equal status, common goals, collaboration, and structural support should predict more favorable outgroup attitudes due to more positive interaction quality perceptions within the intensive longitudinal data.

Once the general contact hypothesis is established within the ESM data, our second main aim is to test our main theoretical proposal that the fulfillment of situational needs is meaningfully related to more positive out-group attitudes following intergroup interactions. As our main proposal is concerned with the mechanisms of successful intergroup contact, we again focus on outgroup interaction reports. Within a multilevel model, we expect interactions that are higher in situational need fulfillment to be perceived as more positive, and as a result, these interactions also predict more positive outgroup attitudes. We also expect the needs mechanism to work at least as well as Allport’s conditions. We particularly expect part of Allport’s contact conditions to be a static set of situational needs so that the situational need fulfillment should explain some of the same variance in out-group attitudes.

**Hypothesis 3:** Based on our proposal, intergroup interactions with higher situational need fulfillment should predict more favorable outgroup attitudes due to more positive interaction quality perceptions within the intensive longitudinal data. We also expect situational need fulfillments to work at least as well as Allport’s optimal contact conditions in predicting out-group attitudes.

Our third main aim is to ensure that our results are robust, stable, and ecologically valid. To test the robustness of our need-fulfillment mechanism, we test whether the need mechanism is indeed specific to out-group interactions and whether the process could be explained by a smaller set of fundamental psychological needs instead. We additionally, assess the need fulfillment mechanism in predicting individual well-being benefits and check whether different types of needs or interactions change the main results. We present the full robustness analyses in Appendix B. To test the stability and reliability of our results, we utilize forest plots and meta-analytic estimates for our main analyses. To assess the embeddedness of our situational needs, we use an exploratory topic model for the participants’ free-text entries and compare the extracted content topics with themes commonly found within the motivational literature.

Before turning to individual studies, we would like to address a number of conceptual, practical, and methodological considerations. One key decision for our studies has been to focus on the minority experience during the contact. While the same mechanisms should hold for the experience of members in high-power groups, there is substantially more research available that focuses on the experience of the majority group, and minority perspectives are historically often understudied (e.g., Dovidio et al., 2017). At the same time, however, minority groups are often underprivileged and research is direly needed to understand the more prevalent experiences of stress and health issues among minorities (e.g., Alvidrez et al., 2019).

A second non-trivial aspect of translating the intergroup contact hypothesis into intensive real-world data was the choice of the outcome variable. For our main analyses, we chose outgroup attitudes—the positive or negative evaluation of the other group. We chose out-group attitudes mainly because they are the most common outcome considered within the intergroup contact literature (e.g., Paolini et al., 2021; Pettigrew & Tropp, 2006). As the methodology is relatively new to the field, we sought to first replicate (and then extend) the most reliable effects of the contact hypothesis within the ESM data. Out-group attitudes are, however, not without controversy, especially for minority group members. Positive out-group attitudes can increase harmony and reduce the willingness to support social change among the disadvantaged in some cases—even in the face of injustice (e.g., Dixon et al., 2012; Saguy et al., 2009). While a recent review found the effect to be less conclusive for longitudinally collected data and less consistent for positive interactions (rather than interactions generally), the backfire effect remains an important possibility for the present data (see Reimer & Sengupta, 2023). To ensure at least a direct benefit to the minority group members, we also assess the effect of the need fulfillment mechanism on well-being as the dependent variable as part of our robustness analyses below.

In terms of methodological considerations, it is important to note we tested most of our hypotheses using multilevel regression models, where measurement occasions (level 1) were nested within participants (level 2). This approach is tolerant to missing data and uneven case numbers within participants. Furthermore, we use a hierarchical modeling approach and report the final model in-text (Snijders & Bosker, 2012, for the full modeling process, see Supplementary Material A). Second, statistical power estimations for intensive longitudinal—and multilevel models are notoriously difficult due to the complex covariance structures. However, our participant—and measurement numbers are among the largest sample sizes found within the intensive longitudinal literature (e.g., aan het Rot et al., 2012). In addition, power simulations after the first study showed that our data was sufficiently powered for even small effect sizes (see Supplemental Material B). In particular, we found that even our smallest effects of interest would be detectable with 22 participants and 24 measurements per person (assuming the effect sizes of Study 1 and focusing on a power of .8 with a .05 alpha level; see Supplemental Material B for the full analyses). We only increased the participant sample sizes in Studies 2 and 3 to allow for between-participant effects across studies and more complicated trajectory analyses, which are not necessary for the hypotheses tested here.

Finally, for our most comprehensive study (Study 3) we pre-registered both the hypotheses and the analysis plan (available at Kreienkamp et al., 2021). All studies received ethical approval from the University of Groningen, and none of the data have been published elsewhere. The detailed hypotheses and analysis plan are available in Appendix A. The full surveys, code, and materials are available in our open science repository (including a complete codebook; Kreienkamp et al., 2022a, 2022b) In addition, the fully annotated analyses are available in Supplementary Materials A, B, and C.

## Study 1

Based on our main hypotheses, our first study aimed to specifically test the general contact hypothesis, the influence of situational need fulfillment, and perceived interaction quality during intergroup contacts. To this aim, we recruited recent migrants to the Netherlands for an intensive longitudinal survey. Data were collected from May 5th through June 6th, 2018 (and all participants started the study within the first 2 days). Correlations and descriptive statistics of the included variables are available in Tables 1 and 2 (full data description is available in Online Supplementary Material A).

**Table 1.** Full Sample: Correlation Table and Descriptive Statistics.

Variable	Correlations					Descriptives			
	Sit. need	Quality	Attitudes NL	Allport	Grand Mean	Between *SD*	Within *SD*	ICC(1)	ICC(2)
Study 1									
Sit. Need		.37***	.11***		77.95	14.68	20.83	0.29	0.96
Quality	.46*		.50***		67.00	9.26	17.43	0.23	0.83
Attitudes NL	−.18	.19			71.49	12.91	8.11	0.70	0.99
Study 2									
Sit. Need		.21***	.08***		84.87	9.17	20.33	0.15	0.89
Quality	.61***		.09***		74.51	11.24	16.59	0.29	0.92
Attitudes NL	.03	.02			67.26	18.64	9.40	0.80	0.99
Study 3									
Sit. Need		.37***	.10***	0.42***	83.57	8.02	17.14	0.18	0.92
Quality	.51***		.08***	0.56***	76.62	12.42	16.98	0.34	0.96
Attitudes NL	.06	.08		0.23	64.77	14.37	10.88	0.66	0.99
Allport	.64***	.54***	.04*		86.74	7.08	11.87	0.25	0.95
Across Studies									
Sit. Need		.29***	.10***		83.66	9.75	19.35	0.19	0.92
Quality	.52***		.11***		74.47	11.75	16.82	0.31	0.94
Attitudes NL	.04	.02			66.88	16.76	9.81	0.75	0.99

*Note.* Sit. = Situational, Attitudes NL = Attitudes towards the Dutch, ICC = Intraclass Correlation Coefficient; Upper triangle: Within-person correlations; Lower triangle: Between-person correlations.

* *p* < .05. **p < .01. ***p < .001.

**Table 2:** Intergroup Contact Sample: Correlation Table and Descriptive Statistics.

Variable	Correlations					Descriptives			
	Sit. need	Quality	Attitudes NL	Allport	Grand Mean	Between *SD*	Within *SD*	ICC(1)	ICC(2)
Study 1									
Sit. Need		.37***	.27***		82.20	12.42	17.66	0.33	0.90
Quality	.40		.55***		67.00	9.26	18.24	0.23	0.84
Attitudes NL	−.03	.21			72.46	13.62	9.50	0.68	0.98
Study 2									
Sit. Need		.23***	.16***		86.86	11.20	15.87	0.14	0.58
Quality	.23		.29***		67.08	12.54	16.54	0.24	0.73
Attitudes NL	.17	.06			70.41	17.13	9.87	0.72	0.96
Study 3									
Sit. Need		.34***	.24***	0.37***	84.84	9.27	13.00	0.30	0.91
Quality	.52***		.29***	0.56***	71.95	14.97	16.71	0.43	0.95
Attitudes NL	.23	.33**		0.40***	68.24	13.72	11.23	0.63	0.98
Allport	.60***	.44***	.23***		80.87	10.87	12.14	0.42	0.95
Across Studies									
Sit. Need		.30***	.21***		85.65	10.75	15.14	0.26	0.84
Quality	.37***		.32***		68.78	13.31	16.79	0.34	0.89
Attitudes NL	.13	.09			69.86	15.64	10.34	0.69	0.97

*Note.* Sit. = situational, Attitudes NL = Attitudes towards the Dutch, ICC = Intraclass Correlation Coefficient; Upper triangle: Within-person correlations; Lower triangle: Between-person correlations.

* *p* < .05. **p < .01. ***p < .001.

### Methods

#### Participants

After receiving ethical approval from the University of Groningen, we recruited 23 non-Dutch migrants using the local paid participant pool. Participants reported on their interactions for at least 30 days with two daily measures (capturing the morning and afternoon). With this design, we aimed at getting 50-60 measurements per participant (*M* = 53.26, *SD* = 16.72, *total N* = 1,225). This is a common number of measurements found in experience sampling studies and offers sufficient power to model processes within and between participants (e.g., aan het Rot et al., 2012). Participants were compensated for their participation with up to 34 Euros—each two Euros for pre- and post-questionnaire and 50 Eurocents for every experience sampling measurement. The sample consisted of relatively young, educated, and western migrants from the global north (*M_age_* = 24.35, *SD_age_* = 4.73, 19 women, 15 students). The sample accurately describes the largest groups of migrants in the region (see Gemeente Groningen, 2015, for a recent report on the largest migrant groups and see Supplemental Material A for a full overview of the demographic composition, including country of origin).

#### Procedure

The study itself consisted of three main parts, an introductory pre-measurement, the daily experience sampling measurements, and a concluding post-measurement. After giving informed consent, participants filled in an online pre-questionnaire assessing demographics and general information about their immigration. Over the next thirty days, participants were invited twice a day (at 12 pm and 7 pm) to reflect upon their interactions, situational need fulfillments, and current attitudes toward the Dutch outgroup. General compliance was high (85.90% of all invited surveys were filled in).^2^ The response rates were approximately equal during mornings (*n* = 621) and afternoons (*n* = 604) and most measurements were completed within four hours of the invitation. After the final day of experience sampling measurements, participants were invited to fill in a longer post-measurement survey that mirrored the pre-measurement. All key variables for this study were part of the short experience sampling surveys.

### Materials

#### Intergroup Contact

To test the prerequisite effect of intergroup contact, every experience sampling measurement started with the question “*Did you meet a Dutch person this morning [/afternoon]? (In person interaction for at least 10 minutes)*.” Our participants recorded between 2–51 interactions with Dutch outgroup members (*M* = 31.71%, *SD* = 19.88% of the individuals’ experience sampling measurements; 387 of all 1,225 experience sampling responses)Two participants only recorded two experience sampling measurements each and none of these included out-group contacts. These participants are removed from any analyses that focus on out-group contacts.

#### Need Fulfillment

Irrespective of whether participants had an interaction with Dutch people or not, everyone answered a short series of questions on situational need fulfillment. However, whereas participants with interactions reported on the need fulfillment during the interaction, people without interactions with Dutch people judged the past daytime period in general. To assess the fulfillment of needs, we included two types of need measurement: (a) the situational need and (b) general self-determination theory needs.

For the situational need, we asked participants in an open-ended text field: “*What was your most important goal [during the interaction / this morning / this afternoon]?*.” Then, with reference to the text entry, we asked how much this situational need was fulfilled during the interaction or the past daytime period: “*[The interaction / You] fulfilled your goal: [-previous text entry-]*” on a continuous slider scale ranging from strongly disagree (−50) to strongly agree (+50). The self-determination theory need measurements were collected for robustness analyses and are described in Appendix B.

#### Perceived Interaction Quality

To assess ratings of the perceived interaction quality, participants rated the statement “*Overall the interaction was . . .*” on two continuous slider scales measuring pleasantness, from unpleasant (−50) to pleasant (+50) and meaningfulness, from superficial (−50) to meaningful (+50). The items formed a coherent concept within the participants (*r_within_* = .54, *p* < .001). We adapted this from Downie et al. (2008), who validated the approach.

#### Out-group Attitudes

At the end of every experience sampling measurement, we asked all participants about their current attitudes toward the Dutch. To assess the momentary outgroup evaluation we used the common feeling thermometer: “How favorable do you feel towards the Dutch?” (Nelson, 2008). Participants then rated their attitude on a continuous slider scale from “very cold—0” through “no feeling—50” to “very warm—100.” Both the question phrasing as well as the tick labels were consistent with large-scale panel surveys (e.g., DeBell et al., 2010).

### Results

#### Contact Hypothesis

Using a multilevel regression, we find that having an outgroup contact is indeed associated with significantly more positive outgroup attitudes, *b* = 2.48, *t*(1,200) = 4.37, *p* < .001, 95% confidence interval [CI]: [1.37, 3.59], even after controlling for having an interaction with a non-Dutch (which did not relate to outgroup attitudes independently). In addition, while multilevel regressions are generally robust against unequal cell sizes, we correct for inequalities by using centered predictors and reintroducing the means as level two predictors (Yaremych et al., 2021; for full results see Table 3, Figure 2, and Online Supplementary Material A).^3^ Thus, in our first data, we find initial evidence that outgroup contacts show a positive effect on outgroup attitudes within real-life data.

**Table 3.** Intergroup General.

	Study 1		Study 2		Study 3	
Variable	B	β	B	β	B	β
Within Participants [multilevel linear regression] (EffectSize = β)						
(Intercept)	74.12**** [56.74, 91.49]		67.05**** [57.37, 76.73]		60.36**** [46.45, 74.27]	
Out-group Interaction	2.48**** [1.37, 3.59]	1.07 [0.59, 1.54]	2.83*** [1.28, 4.38]	0.11 [0.07, 0.16]	5.57**** [3.90, 7.23]	0.21 [0.15, 0.27]
Non-Out-group Interaction	0.44 [−0.67, 1.55]	0.16 [−0.32, 0.63]	0.02 [−0.76, 0.79]	0.01 [−0.03, 0.05]	0.36 [−0.48, 1.19]	0.02 [−0.02, 0.05]
Out-group Interaction Mean	0.99 [−26.61, 28.59]	1.65 [−4.27, 7.57]	26.53** [8.61, 44.46]	0.00 [−0.03, 0.03]	14.14 [−2.19, 30.48]	0.00 [−0.03, 0.03]
Non-Out-group Interaction Mean	−4.82 [−30.26, 20.63]	−1.08 [−7.02, 4.86]	−8.32 [−22.98, 6.35]	0.00 [−0.03, 0.03]	−2.75 [−20.00, 14.50]	0.00 [−0.03, 0.03]
$R_{marginal}^{2}/R_{conditional}^{2}$	.009 / .722		.050 / .818		.049 / .717	
Between Participants [aggregated linear regression] (EffectSize = η_partial_)						
(Intercept)			68.24**** [65.30,71.18]			
Sum Contact NL			0.51** [0.12, 0.89]; η_partial_ = 0.03			
Average Quality Outgroup Interaction			0.27* [0.03, 0.50]; η_partial_ = 0.05			
Study (1)			2.50 [−4.86, 9.85]; η_partial_ = 0.00			
Study (3)			−2.97 [−7.67, 1.72]; η_partial_ = 0.01			
Sum Contact NL—Average Quality Outgroup Interaction			−0.01 [−0.03, 0.00]; η_partial_ = 0.01			
Sum Contact NL—Study (1)			−0.38 [−1.14, 0.37]; η_partial_ = 0.00			
Sum Contact NL—Study (3)			−0.45 [−0.91, 0.02]; η_partial_ = 0.02			
Average Quality Out-group Interaction X Study (1)			0.07 [−0.76, 0.90]; η_partial_ = 0.00			
Average Quality Out-group Interaction X Study (3)			−0.01 [−0.35, 0.33]; η_partial_ = 0.00			
$R_{marginal}^{2}/R_{conditional}^{2}$			.118 / .076			

*Note.* For the within-participant regressions (upper group), the variables marked “Mean” indicate the re-introduced participant means (level two), which are also standardized across participants.

* *p* < .05. **p < .01. ***p < .001. ****p < .0001.

#### Situational Need Fulfillment

**Figure 1:**
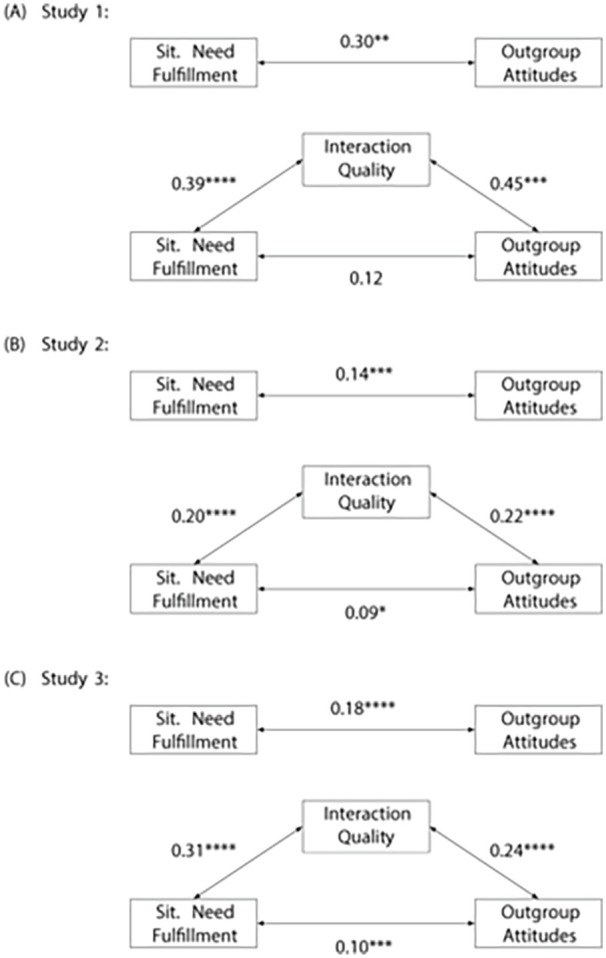
Partial Regression Diagrams of Situational Needs Model Across Studies. *Note.* Coefficients are standardized (partial) regression coefficients. Statistical significance markers are based on the unstandardized regression results (as presented in Table 4). Note that we do not test a mediation model. The diagram only illustrates the included concepts and partial regression parameters. Sit. = situational. *p < .05. **p < .01. ***p < .001. ****p < .0001.

The main proposal of our article is that the success of an outgroup contact might be explained by whether or not the contact fulfilled the person’s situational need. This should, in turn, be reflected in higher perceived contact quality and more positive outgroup attitudes. We sequentially test whether the fulfillment of the situational need during an interaction is (a) related to more positive outgroup attitudes, (b) higher perceived contact quality, and (c) whether the variance explained by the situational need is subsumed by the perceived contact quality if considered jointly. We find that in the multilevel models, the fulfillment of situational needs during out-group contacts was associated with more positive outgroup attitudes, random slopes model; *b* = 0.17, *t*(365) = 2.93, *p* = .004, 95% CI: [0.06, 0.29], and also related to higher perceived contact quality, random intercept model; *b* = 0.37, *t*(365) = 7.73, *p* < .001, 95% CI: [0.28, 0.47]. Moreover, when we consider the influences of situational need fulfillment and contact quality on outgroup attitudes jointly, we find that the two predictors share a large part of the variance explained in out-group attitudes so that perceived contact quality showed a strong effect on out-group attitudes, random slopes model; *b* = 0.24, *t*(364) = 4.33, *p* < .001, 95% CI: [0.13, 0.35], and only little unique variance is still explained by situational need fulfillment, *b* = 0.05, *t*(364) = 0.94, *p* = .348, 95% CI: [−0.05, 0.14], also see Figure 1A. We thus find support for our hypotheses and can conclude that in this data set the fulfillment of situational needs had a significant influence on outgroup attitudes. In addition, this effect seemingly addresses the same variance that is accounted for by perceived contact quality.

## Study 2

The aim of Study 2 is similar to Study 1, as we again test the general contact hypothesis, the influence of situational need fulfillment, and perceived contact quality during intergroup contacts. However, in this second study, we collected a substantially larger sample of international students who recently arrived in the Netherlands and also improved the study design (e.g., pop-up explanations described later). The survey method again offers a large body of ecologically valid data on need satisfaction in real-life intergroup contact situations as these students will likely interact with the Dutch majority outgroup on a daily basis. Data were collected from November 19th, 2018, through January 6th, 2019. Correlations and descriptive statistics of the included variables are available in Tables 1 and 2.

### Method

#### Participants

We recruited 113 international students using a local participant pool. We specifically targeted non-Dutch students who had recently arrived in the Netherlands. Participants reported on their interactions for at least 30 days with two daily measures (capturing the morning and afternoon). With this design, we again aimed at receiving 50 to 60 measurements per participant (*M* = 43.94, *SD* = 15.00, *total N* = 4,965). As with the previous study, this should offer sufficient power to model processes within participants and will lend stronger weight to between-participant results. Participants were compensated for their participation with partial course credits—depending on their participation. The sample consisted of relatively young migrants, who were mostly from the global north (*M_age_* = 20.24, *SD_age_* = 2.12, 84 women). The sample fairly accurately describes the local population of international students (see Supplemental Materials A for additional demographic information).

#### Procedure

The study procedure mirrored the setup of Study 1 and consisted of pre-, experience sampling-, and post-measurements. The participants were invited to experience sampling measurements twice a day (at 12 pm and 7 pm) for 30 days. General compliance was high (70.87% of all invited surveys were filled in). The response rates were approximately equal during mornings (*n* = 2,608) and afternoons (*n* = 2,357). All key variables for this study were part of the short experience sampling surveys.

### Materials

#### Intergroup Contact

To measure intergroup contacts, every experience sampling measurement started with the question “*Did you meet a Dutch person this morning [/afternoon]? (in-person interaction for at least 10 minutes)*.” Participants were additionally offered a pop-up explanation: “With in-person interaction, we mean a continued interaction with another person (potentially in a group) that lasted at least 10 minutes. This interaction should be offline and face-to-face. It should include some form of verbal communication and should be uninterrupted to still count as the same interaction. Any individual interaction can last minutes or hours. If there were multiple interaction partners, we would like you to focus on the person that was most important to you during the interaction.” The participants recorded between 1 and 43 interactions with the Dutch majority people (*M* = 20.70%, *SD* = 17.31% of the individual’s experience sampling measurements; 935 of all 4,965 experience sampling responses).

#### Need Fulfillment

For the situational need, we asked participants in an open-ended text field: “*What was your main goal [during the interaction with -X- / this morning / this afternoon]?*” (where *-X-* was dynamically replaced with the name of the interaction partner). Participants could additionally click on a pop-up explanation:

Your main goal during an interaction can vary depending on the interaction. It could be to connect with friends, to find or provide help, to achieve academic ambitions, work on your fitness, work for a job, or simply to get a coffee, just as well as many other concrete or abstract goals that are important to you in the moment. It really depends on your subjective experience of the interaction. . .

Then, with reference to the text entry, we asked how much this situational need was fulfilled during the interaction or the past daytime period: “*During your interaction with -X- [this morning / this evening] your goal (-previous text entry-) was fulfilled.*” on a continuous slider scale ranging from *strongly disagree* (1) to *strongly agree* (100). See Tables 1 and 2 for descriptive statistics.

#### Perceived Interaction Quality

The ratings of the perceived contact quality were identical to Study 1 (item correlation: *r_within_* = .39, *p* < .001).

#### Out-group Attitudes

As in Study 1, attitudes toward the Dutch majority outgroup were again measured using the feeling thermometer.

### Results

#### Contact Hypothesis

**Figure 2:**
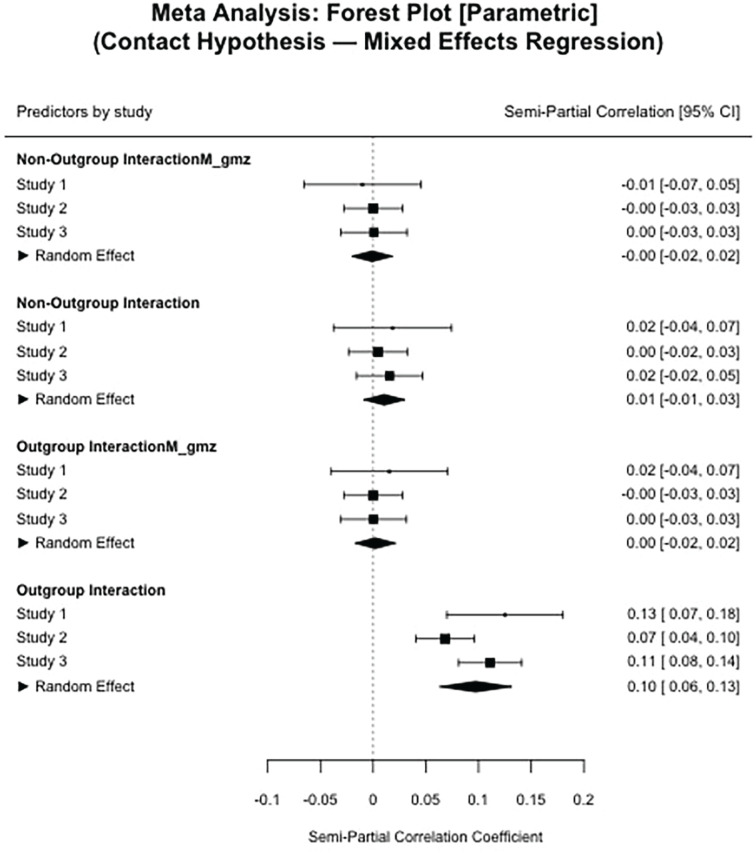
Contact Hypothesis. *Note.* Summary of mixed models results of the contemporaneous contact effects. “M” indicates the re-introduced participant means (level two). “_gmz” indicates that these effects are grand-mean standardized (i.e., across participants). General: Random effects meta-analytic results are presented for completeness. CI = confidence interval.

We tested the most general contact hypothesis as we did for Study 1. We find that having an outgroup interaction is indeed associated with significantly more positive outgroup attitudes within the participants, random slopes model; *b* = 2.83, *t*(4,850) = 3.57, *p* < .001, 95% CI: [1.28, 4.38], even after controlling for having an interaction with a non-Dutch person (which did not relate to outgroup attitudes independently). We again added the participant means back into the model. We find that in this data set participant-level, out-group contact proportions were also a positive predictor of outgroup attitudes, *b* = 26.55, *t*(110) = 2.90, *p* = .004, 95% CI: [8.61, 44.46]. The relative number of non-out-group interactions showed no such effect (for full results see Table 3, Figure 2, as well as Online Supplementary Material A). Thus, in our second data set, we also find that outgroup contacts show a positive effect on out-group attitudes at the moment. We additionally find an average between-participant effect of the relative number of interactions participants had.

#### Situational Need Fulfillment

We again sequentially tested the situational need model as we did for Study 1. We find that in the multilevel models, the fulfillment of situational needs during outgroup contacts was associated with more positive outgroup attitudes, random slopes model; *b* = 0.13, *t*(826) = 4.18, *p* < .001, 95% CI: [0.07, 0.19], and also predicted higher perceived interaction quality, random slopes model; *b* = 0.29, *t*(826) = 5.43, *p* < .001, 95% CI: [0.19, 0.40]. In addition, if we consider the influences of situational need fulfillment and interaction quality on out-group attitudes jointly, we again find that much of the explained variance is shared by the predictor variables so that perceived interaction quality remains a strong predictor, random slopes model; *b* = 0.16, *t*(825) = 5.93, *p* < .001, 95% CI: [0.11, 0.21], and only little unique variance is still explained by situational need fulfillment, *b* = 0.06, *t*(825) = 2.47, *p* = .014, 95% CI: [0.01, 0.11]; also see Figure 1B and Table 4 for full results. These results are consistent with the results in Study 1. We, thus, find support for our hypotheses that the fulfillment of situational needs had a significant influence on perceived interaction quality and outgroup attitudes.

**Table 4.** Theoretical Test: Situational Need Fulfillment and Allport’s Conditions.

	Quality				Attitude			
Variable	B	β	B	β	B	β	B	β
Study 1: Needs								
(Intercept)	67.16**** [62.99, 71.34]		72.53**** [66.81, 78.26]		72.50**** [66.74, 78.26]			
Situational Need	0.37**** [0.28, 0.47]	0.39 [0.30, 0.48]	0.17** [0.06, 0.29]	0.30 [0.18, 0.41]	0.05 [-0.05, 0.14]	0.12 [0.00, 0.24]		
Quality					0.24*** [0.13, 0.35]	0.45 [0.31, 0.59]		
$R_{marginal}^{2}/R_{conditional}^{2}$	.103 / .333		.030 /.753		.068 /.858			
Study 2: Needs								
(Intercept)	67.46**** [65.17, 69.76]		70.71**** [67.55, 73.87]		70.69**** [67.52, 73.85]			
Situational Need	0.29**** [0.19, 0.40]	0.20 [0.10, 0.31]	0.13*** [0.07, 0.19]	0.14 [0.06, 0.22]	0.06* [0.01, 0.11]	0.09 [0.01, 0.17]		
Quality					0.16**** [0.11, 0.21]	0.22 [0.13, 0.30]		
$R_{marginal}^{2}/R_{conditional}^{2}$	.054 /.343		.012 /.750		.024 /.780			
Study 3: Needs								
(Intercept)	72.20**** [68.71, 75.70]		68.32**** [65.10, 71.54]		68.32**** [65.10, 71.54]			
Situational Need	0.45**** [0.36, 0.54]	0.31 [0.24, 0.37]	0.19**** [0.12, 0.27]	0.18 [0.11, 0.24]	0.12*** [0.06, 0.19]	0.10 [0.04, 0.16]		
Quality					0.16**** [0.11, 0.20]	0.24 [0.18, 0.31]		
$R_{marginal}^{2}/R_{conditional}^{2}$	.063 /.517		.019 /.680		.039 /.706			
Study 3: Allport								
(Intercept)	72.23**** [68.75, 75.72]		68.36**** [65.16, 71.56]		68.36**** [65.14, 71.58]			
Allport	0.62**** [0.49, 0.74]	0.40 [0.33, 0.47]	0.22**** [0.15, 0.29]	0.21 [0.14, 0.27]	0.11** [0.05, 0.18]	0.11 [0.05, 0.17]		
Quality					0.16**** [0.11, 0.21]	0.23 [0.16, 0.29]		
$R_{marginal}^{2}/R_{conditional}^{2}$	.111 /.591		.024 /.676		.041 /.699			
Study 3: Needs & Allport								
(Intercept)							68.33**** [65.05, 71.61]	
Situational Need							0.13**** [0.08, 0.17]	0.12 [0.05, 0.18]
Allport							0.17*** [0.09, 0.24]	0.17 [0.11, 0.23]
$R_{marginal}^{2}/R_{conditional}^{2}$							.086 / —	

* *p* < .05. **p < .01. ***p < .001. ****p < .0001.

### Study 3

The aim of this final study is to extend the previous studies by additionally testing Allport’s conditions in an intensive longitudinal design and to compare the predictive powers of Allport’s conditions and the situational need fulfillment. For this study, we specifically recruited international medical students because they represent a particular group of migrants who face structural requirements to integrate and interact with Dutch majority outgroup members on a daily basis. As part of their educational program, the migrants are required to take language courses and interact with patients as part of their medical internships and medical residency. The intensive longitudinal survey method again offers a large body of ecologically valid data on need satisfaction in real-life intergroup contact situations. Data were collected from November 8th, 2019, to January 10th, 2020. The full pre-registration is available at Kreienkamp et al. (2021). Correlations and descriptive statistics of the included variables are available in Tables 1 and 2.

#### Method

##### Participants

We recruited 71 international medical students using contacts within the University Medical School. We specifically targeted non-Dutch students, who had recently arrived in the Netherlands. Participants reported on their interactions for at least 30 days with two daily measures (capturing the morning and afternoon). With this design, we aimed at getting 50 to 60 measurements per participant (*M* = 57.85, *SD* = 20.68, *total N* = 4,107). As with the previous studies, this offered sufficient power to model processes within participants. Participants were compensated in the same manner as during Study 1. The sample consisted of relatively young migrants (*M_age_* = 22.68, *SD_age_* = 3.10, 59 women). The sample fairly accurately describes the local population of young international medical professionals (see Supplemental Materials A for additional demographic information).

##### Procedure

The study procedure mirrored the setup of Studies 1 and 2 and included the same pre-, experience sampling-, and post-measurement phases. The participants were invited to experience sampling measurements twice a day (at 12 pm and 7 pm) for at least 30 days. General compliance was high (85.92% filled in at least 31 experience sampling surveys or more). The response rates were approximately equal during mornings (*n* = 2,092) and afternoons (*n* = 2,015). All key variables for this study were part of the short experience sampling surveys.

#### Materials

##### Intergroup Contact

The measurement of intergroup contacts was identical to Study 2. The participants recorded between 1 and 71 interactions with Dutch out-group members (*M* = 42.22%, *SD* = 19.96% of the individuals’ experience sampling measurements; 1,702 of all 4,107 experience sampling responses).

##### Need Fulfillment

The measurement of the situational need and its fulfillment was identical to Study 2.

##### Allport’s Conditions

We measured how much each of the interactions fulfilled Allport’s conditions of optimal contact using a common short scale comprised of four attributes (Al Ramiah & Hewstone, 2012; Islam & Hewstone, 1993; Voci & Hewstone, 2003). In particular, we asked participants to rate how much the interaction had equal status (“*The interaction with [name interaction partner] was on equal footing (same status)*”), a common goal (“*[name interaction partner] shared your goal ([free-text entry interaction key need])*”), support of authorities (“*The interaction with [name interaction partner] was voluntary*”), and intergroup cooperation (“*The interaction with [name interaction partner] was cooperative*”). We create a mean-averaged index of Allport’s conditions in response to past findings indicating that the conditions are best conceptualized jointly and as functioning together rather than as fully independent factors (Pettigrew & Tropp, 2006, p. 766). For full psychometric information see Online Supplementary Material A.

##### Perceived Interaction Quality

The ratings of the perceived contact quality were identical to Study 1 and Study 2 (item correlation: *r_within_* = .46, *p* < .001).

##### Perceived Interaction Quality

The ratings of the perceived interaction quality were identical to Study 1.

##### Outgroup Attitudes

Attitudes toward the Dutch majority outgroup were again measured using the feeling thermometer, as in Studies 1 and 2.

### Results

#### Contact Hypothesis

In a multilevel regression, we find that having an outgroup interaction was again associated with significantly more positive out-group attitudes within the participants, random slopes model; *b* = 5.57, *t*(3,834) = 6.52, *p* < .001, 95% CI: [3.90, 7.23], even after controlling for having a non-Dutch interaction (which did not relate to out-group attitudes independently; for full results see Table 3 and Figure 2). Thus, in our third data set, we find that the within-person contemporaneous effect of intergroup contact was consistent across all three studies.

#### Situational Need Fulfillment

We tested the situational needs model analogous to the previous studies. We find that the fulfillment of the situational need during outgroup contacts was associated with more positive outgroup attitudes, random slopes model; *b* = 0.19, *t*(1,601) = 5.29, *p* < .001, 95% CI: [0.12, 0.27], and also predicted higher perceived interaction quality, random slopes model; *b* = 0.45, *t*(1,605) = 9.32, *p* < .001, 95% CI: [0.36, 0.54]. In addition, once we consider the influences of situational need fulfillment and interaction quality on outgroup attitudes jointly, we find that perceived interaction quality is a substantially stronger predictor, random slopes model; *b* = 0.16, *t*(1,600) = 7.03, *p* < .001, 95% CI: [0.11, 0.20], and the unique variance explained by situational need fulfillment was roughly half of its original effect size, *b* = 0.12, *t*(1,600) = 3.61, *p* < .001, 95% CI: [0.06, 0.19]; also see Figure 1C and Table 4 for full results. As with the previous two studies, these results indicate that in this data set outgroup attitudes were significantly predicted by the fulfillment of situational needs and the results suggest that this explained variance is shared with perceived interaction quality.

#### Allport’s Conditions

We tested the impact of Allport’s conditions in the same manner as we tested our situational needs model. In the multilevel models, we find that the fulfillment of Allport’s Conditions during outgroup contacts was associated with more positive out-group attitudes, random slopes model; *b* = 0.22, *t*(1,601) = 5.86, *p* < .001, 95% CI: [0.15, 0.29], and also predicted higher perceived interaction quality, random slopes model; *b* = 0.62, *t*(1,605) = 9.60, *p* < .001, 95% CI: [0.49, 0.74]. Moreover, when we considered the influences of Allport’s Conditions and interaction quality on outgroup attitudes jointly, we found that perceived interaction quality was a substantially stronger predictor, random slopes model; *b* = 0.16, *t*(1,600) = 6.56, *p* < .001, 95% CI: [0.11, 0.21], and the unique variance explained by Allport’s Conditions was less than half of its original effect size, *b* = 0.11, *t*(1,600) = 3.54, *p* < .001, 95% CI: [0.05, 0.18]; also see Table 4. These results indicate that in this data set the fulfillment of Allport’s conditions had a significant influence on out-group attitudes and this effect is likely related to the effect of perceived interaction quality.

#### Compare Fulfillment of Situational Need and Allport’s Conditions

To test whether Allport’s conditions or the situational need fulfillment were better at predicting outgroup attitudes, we first assessed relative model performance indices (i.e., Akaike information criterion, and Bayesian information criterion), and then considered the two predictors in a joint model to see whether the two approaches predicted the same variance in out-group attitudes. When comparing the model selection indices, we found that the fulfillment of the situational need indeed performed slightly better than the model using Allport’s conditions (*AIC_SituationalNeed_* 12,632.02 < 12,651.59 *AIC_Allport_*, and *BIC_SituationalNeed_* 12,664.55 < 12,684.12 *BIC_Allport_*). In addition, when considering the predictors jointly, we find that both significantly predict out-group attitudes with similar-sized regression parameters, random slopes model; Allport’s Conditions: *b* = 0.16, *t*(1,600) = 4.92, *p* < .001, 95% CI: [0.09, 0.24], Situational Need: *b* = 0.14, *t*(1,600) = 3.85, *p* < .001, 95% CI: [0.08, 0.17]; also see Table 4. This indicates that, although both Allport’s conditions and the situational need fulfillment seem to (in part) relate to perceived interaction quality, they explain different aspects of the variance in outgroup attitudes and do not constitute one another.

## Stability, Robustness, and Embeddedness Across Studies

Beyond the individual results of the three studies, we conducted a number of additional analyses to test the broader cross-study claims, account for alternative models, and contextualize our results. Jointly, these stability, robustness, and embeddedness analyses seek to strengthen our confidence in the results.

### Stability

We ran two analyses that tested the stability of our results. We first assess the consistency of the results reported in the three studies and use a meta-analytic approach to gauge the general effect sizes. The second stability analysis we conduct seeks to assess the extent to which the within-person contemporaneous effects extend to an aggregated version that mirrors the many cross-sectional recall studies.

#### Consistency

**Figure 3:**
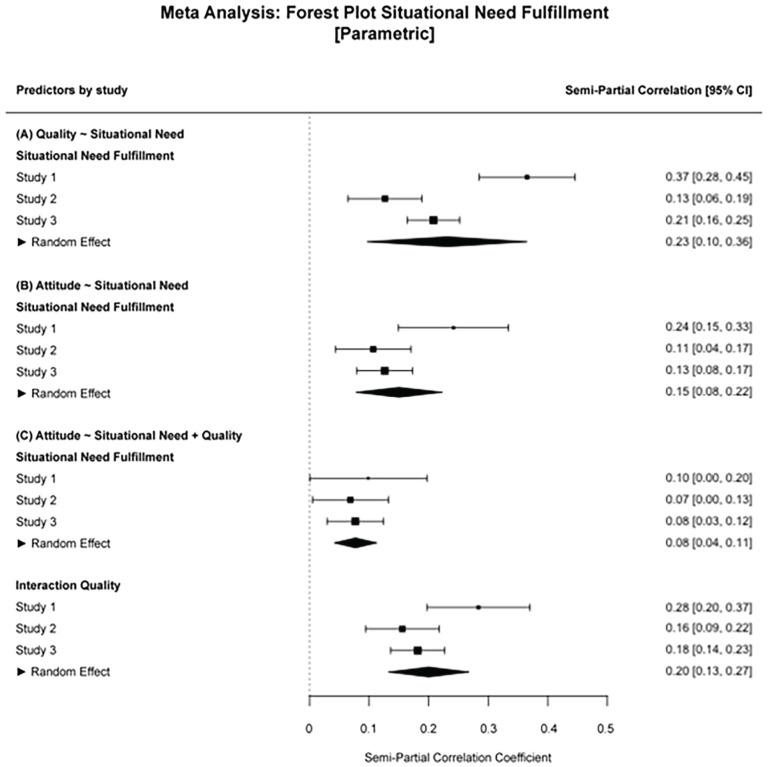
Situational Need Fulfillment. *Note.* (A) Situational Need Fulfillment predicting Interaction Quality. (B) Situational Need Fulfillment predicting Outgroup Attitudes. (C) Situational Need Fulfillment and Interaction Quality predicting Outgroup Attitudes. General: Random effects meta-analytic results are presented for completeness. CI = confidence interval.

We first assessed the stability of our main analyses across the three studies. Plotting the effect sizes of each parameter of interest in a forest plot, as well as the average meta-analytic effect, shows that for the basic contact hypothesis test out-group contact had a strong and consistent effect on out-group attitudes. Interactions with non-out-group members consistently had no meaningful effect on outgroup attitudes (Figure 2). While this would be expected from the general intergroup contact literature, this is not a trivial finding. Being among the first to assess the contact hypothesis using real-life intensive longitudinal data, we extend cross-sectional findings to individual-level assessments. When looking at the fulfillment of situational needs during intergroup contacts, we find that the motivational mechanism is consistently a meaningful predictor of interaction quality perceptions and out-group attitudes (see [A] and (B) in Figure 3). We also see that the effect of situational need fulfillment on out-group attitudes is strongly reduced when modeled together with interaction quality perceptions, supporting our assertion that interaction quality and need fulfillment share the same variance explained in out-group attitudes (see [C] in Figure 3). Note that this joint effect is not meant to resemble a mediation analysis. Particularly, since the data is non-causal, and because multicollinearity and potential third variables could result in similar results.

#### Aggregated Analysis

A second stability test is checking whether the within-person effects translate to a broader aggregated between-subjects effect, which would mirror common cross-sectional practices. During the main analyses, we have thus far shown that participants held more positive outgroup attitudes following intergroup contacts and that perceived interaction quality was associated with more positive outgroup attitudes following an intergroup contact. We have, however, not brought the two quantity and quality elements of the contact hypothesis together in a single analysis. To jointly test the effects of contact frequency and average interaction quality, we ran a linear regression model where average outgroup attitudes were predicted by the number of interactions and the average interaction quality ratings of all participants across the three studies. We did so while controlling for the possible effects of study-specific differences. To include the study membership as a control variable, we used the student sample (Study 2) as the reference group because it was both the largest and the most homogeneous study. Looking at the overall model, we found that the model predicted 11.84% of the variance in average out-group attitudes, *F*(9, 189) = 2.82, *p* = .004, *R*^2^ = .12. Looking at the individual effects, we found that only the number of outgroup interactions has a clear association with average outgroup attitudes, *b* = 0.51, *t*(189) = 2.61, *p* = .010, 95% CI: [0.12, 0.89]. The average interaction quality perceptions had a much smaller effect, *b* = 0.27, *t*(189) = 2.24, *p* = .026, 95% CI: [0.03, 0.50], and importantly we found no interaction effect at all. In short, the effect of interaction frequency did not depend on the average interaction quality.

### Robustness

To check for spurious relationships, we test three additional models. These three models assess whether the effect is indeed specific to intergroup contact, whether the need fulfillment effect is affected by whether the contact was planned or accidental, and whether the motivational mechanism also holds for direct well-being benefits for the minority members. We describe the full methods and results for the robustness analyses in Appendix B. We use a two-staged analysis approach for the robustness analyses. We first test the model “globally”—across the three studies—in a three-level multilevel regression (i.e., measurements nested within participants, and participants nested within studies). Only in a second step do we check for study-specific idiosyncrasies.

#### Contact Specific

To ensure that the situational need fulfillment is out-group contact specific, we return to the full sample of intensive longitudinal measurements and test whether there is an interaction effect of outgroup contact (vs. no outgroup contact) and situational need fulfillment. We expected that the effect of situational need fulfillment on outgroup attitudes is specific to out-group interactions and not merely due to a more need-fulfilled life in general. Both in the global test, as well as in the individual studies, we consistently find a significant and meaningful interaction effect of outgroup interaction and situational need fulfillment, indicating that situational need fulfillment is specifically a powerful predictor of outgroup attitudes during intergroup contacts. When assessing the individual studies independently, we additionally find a strong main effect of contact as well as a smaller main effect of situational need fulfillment, qualifying the contact-specific relationship (see Appendix B for full results). In sum, we thus find that at the effect of situational need fulfillment on out-group attitudes is particularly important for outgroup interactions (rather than need fulfillment in general).

#### Interaction Intent

To test whether the need mechanism was affected by whether the interaction was accidental or planned we ran an exploratory moderation analysis using the participants’ ratings of how much they perceived the interaction as “accidental.” It should be noted that, to keep the ESM surveys short, we asked our participants to focus on most of the important interaction (i.e., “*The following questions will be about the interaction you consider most significant.*”; emphasis as in original). This was also reflected in a relatively low mean and consistent right skew of the “accidental” item. Nonetheless, there remained a substantial variance in the item and we continued with the moderation analysis. We found that both in the overall test and in all studies individually situational need fulfillment remained a strong predictor of outgroup attitudes, even when accounting for differences in whether the interaction was accidental (rather than planned). Moreover, in none of the analyses did we see a moderation effect of interaction intent nor did we find a main effect of interaction intent. There is, thus, consistent evidence that the need fulfillment mechanism was not meaningfully different for more accidental interactions.

#### Well-Being Outcome

Given the well-established criticism that more positive outgroup attitudes might not always be beneficial for minority group members (e.g., Reimer & Sengupta, 2023), we conducted an additional exploratory analysis assessing the effect of need-fulfilling outgroup interactions on reported well-being. Experienced well-being is a common indicator of health and an important life quality measurement in itself, especially for migrants or other minority groups (e.g., Bhugra et al., 2011). We find that the results with well-being as the outcome variable mirror our main results in effect size and interpretation. The results are consistent across and within studies and, thus, add weight to the importance of considering the need fulfillment experiences when it comes to outgroup interactions.

### Embeddedness

To embed our results further, we also considered the content and types of needs that participants reported during the study. For these embeddedness analyses, we first assessed the self-reported motives of the participants and then also controlled the commonly considered self-determination theory needs.

#### Contact Need Content

We used the qualitative data from the participants’ self-identified situational needs to contextualize the results of our main analysis. However, because our participants jointly reported on thousands of intergroup contacts, it would not have been feasible to analyze these qualitative responses in a traditional qualitative content analysis. We instead relied on recent machine learning advances within the natural language processing domain. For our analysis, we used the Bidirectional Encoder Representations from Transformers (BERT) language model. BERT was developed by Google in 2018 and today forms a key element of many natural language processing workflows. In its essence, BERT is a framework that allows users to codify every word in relation to every other word within a large set of documents. We extracted 47 topics from the 2,983 interaction goal free-text entries (after duplicate removal)—a relatively large number of topics. The higher number of topics allowed us to retain more of the smaller topics and leaves a relatively low number of 308 free-text entries unclassified (10.33%). A full write-up of the topic modeling process is available in Online Supplemental Material C.

In terms of the content of the topics, we find that a number of topics are primarily task-oriented, where participants hope to increase their study, research, presentation, or work performance. Opposing the task- and work-oriented needs are a wide variety of leisure-related needs wishes, like relaxation and entertainment. In addition, some clusters were primarily relationship-oriented, so that participants sought contact with outgroup members for intimate and casual social contact in itself. Similarly, socializing and celebrations were also explicit social needs (incl., parties). This also included a subtopic of spiritual, religious, and otherwise transcendental needs (incl. meditation, prayer, religious services). Among the leisure-oriented topics was also a set of contact goals that were specifically migration-specific (e.g., wish to learn about culture, politics, and language) or were concerned with informational needs more generally (e.g., seeking answers, bureaucratic information). A similar set of topics was specifically geared toward a wish to experience cultural products (e.g., music, theater, and food) or had travel-related goals in their interactions with the majority group members. In sum, almost all extracted topics fall into broader or narrower need concepts that are commonly discussed within the need fulfillment literature (e.g., Orehek et al., 2018) and offer insight into a core aspect of the migration experience that has remained broadly under-explored (Kreienkamp et al., 2023).^4^

#### Need Types

To ensure that our results are not impacted by differences in the reported goals and motives, we additionally coded the topics we extracted during the topic modeling on two dimensions of how much they reflect a practical and a psychological goal-directedness. We chose practical and psychological needs specifically as our dimensions to mirror our instructions to the participants and to account for differences in the types of needs that participants commonly reported. With practical motives, we refer to specific, tangible goals or tasks that participants aimed to accomplish during the interaction. These instrumental goals are usually observable, concrete, and often centered on external outcomes, such as acquiring resources, completing tasks, and addressing immediate challenges (e.g., Oduntan & Ruthven, 2019). With psychological motives, we refer to underlying motives or desires that are more abstract and relate to personal fulfillment and well-being. In contrast to practical needs, psychological needs delve into the subjective and internal aspects of human experiences. These needs pertain to emotions, social connections, and cognitive processes, reflecting individuals’ quest for personal growth, well-being, and thriving in social relationships (e.g., Dweck, 2017). Note that with this approach any particular motive can include a practical and/or psychological goal-directedness but can also be classified as not having any goal at all. See Supplemental Materials D for the detailed coding protocol, including instructions for the coders. We found high interrater reliability for the two independent coders, who coded all 47 topics. The codings revealed considerable variance, which allowed us to add the two dimensions back to each of the reported interaction motives and assess the effects of the goal-directed character.

We found that neither in the overall analysis nor within any of the individual studies did different types of motives impact the positive effect of situational need fulfillment on interaction quality perceptions and out-group attitudes. Situational need fulfillment ratings remained the only reliable predictor of out-group attitudes when controlling for practical and psychological goal-directedness as well as their interaction effect with the need fulfillment itself. This result underscores the importance of the experience of perceived need fulfillment (i.e., the perception that one got what one needed), rather than the type of need or the content of the need (i.e., the exact motive).

#### Specific Psychological Needs

Finally, to ensure that a much simpler model of three fundamental psychological needs might not account for the same effect, we compared our situational need fulfillment measurement to the commonly studied self-determination theory needs (autonomy, relatedness, and competence). Within the overall model, we found that across the studies situational need fulfillment remained a strong predictor of out-group attitudes, even after controlling for the three self-determination theory needs. In addition, within this overall analysis, none of the self-determination theory needs independently predicted out-group attitudes to a statistically significant extent (despite a similar effect size of relatedness). When looking at the individual studies, we again saw that situational need fulfillment remained a consistent predictor of out-group attitudes. However, across all three studies the fulfillment of relatedness motives also emerged as a consistent predictor of out-group attitudes. In addition, in the larger Studies 2 and 3 competence fulfillment was also related to more positive outgroup attitudes. None of the autonomy fulfillment effects reached statistical significance. In short, we find that across our samples, relatedness fulfillment (and to a smaller extend competence fulfillment) are instrumental in understanding when an outgroup contact leads to more positive outgroup attitudes. Importantly, even when considering these effects situational need fulfillment remains a strong and consistent predictor of out-group attitudes.

Overall, we find that our situation need fulfillment model is consistent across samples and contexts, and that the need fulfillment effect is robust to a wide variety of alternate explanations.

## Discussion

The main aim of this project was to test the basic tenets of the intergroup contact hypothesis and Allport’s optimal conditions in real-life intensive longitudinal data as well as to test whether the fulfillment of situational needs meaningfully predicts positive interaction perceptions and outgroup attitudes.

When considering the results of the three studies jointly, we found mixed results for the basic intergroup contact hypothesis. To replicate the two common approaches to the contact hypothesis, we looked at both the within-person effects of individual outgroup interactions (mimicking the analysis of lab studies) and the joint effect of interaction frequency and average interaction quality between participants (mimicking the cross-section literature). For the effect of individual interactions, we find that having an outgroup interaction (vs. not having an interaction) was associated with more favorable outgroup attitudes. Similarly, we find that within outgroup interactions, the interaction quality was meaningfully associated with more favorable outgroup attitudes. Yet, considering interaction frequency and average interaction quality jointly was only possible on the aggregated between-participant level. Surprisingly, here we found independent effects of interaction frequency and average interaction quality ratings but no interaction effect. The absence of this aggregate effect could underline the fact that cross-sectional retrospective data might be misleading because (a) it presents a mixture of within and between-subjects effects (Hamaker et al., 2020) or (b) suffers from recall biases (e.g., where times with no out-group interactions are undervalued by participants during the retrospective evaluations).

Interestingly, this effect is also inconsistent with the observations and theorizing of MacInnis and Page-Gould (2015), who observed that individual interactions showed negative effects on intergroup relations and the aggregate of past intergroup contacts showed positive effects on intergroup relations. There are, however, two important differences in our data compared with the past literature assessed by MacInnis and Page-Gould. First, what MacInnis and Page-Gould, called the intergroup interaction literature, has particularly focused on artificial lab studies where study participants meet a stranger from the out-group. In our real-world data, such synthetic and controlled interactions are arguably less relevant. Second, what MacInnis and Page-Gould called the intergroup contact literature, has looked at long-term recall self-reports—where participants are asked to recall the quantity and average quality of intergroup contacts over the past month or year (MacInnis & Page-Gould, 2015). The mental aggregation of such long-term recall surveys is substantially different from the aggregation we did based on the close-to-event reports (Shiffman et al., 2008). To truly compare our results to MacInnis and Page-Gould (2015) theorizing, future studies should, thus, also collect a long-term recall report that mirrors the questions asked during intergroup contact studies.

Our results should also be considered within the emergent literature of panel studies testing the within-person effects of contact on a number of outcomes. Such studies have a different level of resolution and time scale, as they usually collect three to five waves in multi-week, -month, or -year intervals. Importantly to our results, several recent studies have predominantly found a lack of within-person effects in the context of group affiliations and dynamics over varying timescales. Specifically, a number of studies reported no significant within-person effects for either minority or majority group members across intervals ranging from 2 to 6 months to multi-year assessments, with a focus on outgroup solidarity and attitudes (Bracegirdle et al., 2023; Friehs et al., 2023; Özkan et al., 2023; Sengupta et al., 2023). However, contrasting this trend, Górska and Tausch (2023) discovered a within-effect for the majority, indicating that cross-group friendships over three 2-week intervals were associated with future collective action intentions within participants. These studies again highlight that we need studies that bridge the gap between daily close-to-event measurements of natural interactions and longer-term recall studies, even when these longer-term recalls are collected over multiple measurement occasions.

It should also be noted that the inconsistencies with past research might in part be a data artifact (e.g., because most people reported substantially more measurements during which they did not have an outgroup interaction). There is also a possibility that statistical power was a concern given our sample was relatively small with 207 participants and the effect of interest is an amplification interaction effect. However, if we assume an effect size of *r* = −.21 for the effect of positive intergroup contacts (see Pettigrew & Tropp, 2006) our sample size should be at the threshold of 80% power with a .05 alpha level (sensitivity analysis in G*Power: *f* ^2^ *=* 0.04)—even more so if we consider the higher quality data we get from aggregating many real-world reports with less recall bias.

Next, using the data from our third study, we find that Allport’s conditions are related to higher interaction quality perceptions and more positive outgroup attitudes. When we consider interaction quality and Allport’s conditions jointly, we find that interaction quality ratings assumed a larger part of the shared variance in outgroup attitudes. We thus find first evidence that Allport’s conditions of optimal contact are also relevant to the daily interactions recent migrants have in their interactions with majority group members.

Finally, when looking at the results regarding the importance of situational need fulfillment, we find that in all three intensive longitudinal data sets, the fulfillment of situational needs during intergroup contacts predicts higher interaction quality perceptions, more positive outgroup attitudes as well as higher well-being. We also find that in all three studies, need fulfillment and perceived interaction quality likely shared a large part of the variance they explained in outgroup attitudes (when considering partial regression coefficients in a joint model). We would like to reiterate here that we specifically did not seek to test a mediation-style model. Although the shared explained variance was predicted in our pre-registration based on a theoretical model, and the pattern was stable across the three studies, it is important to note that the data is none-causal, and the effect might alternatively be driven by an unobserved third variable or by multicollinearity.

We, additionally, find that need fulfillment is an important predictor even when taking basic fundamental psychological needs or Allport’s conditions into account. In fact, our situational needs measure predicted outgroup attitudes at least as good as Allport’s conditions and consistently explained more variance than commonly measured psychological needs. In most cases, the situational need even took over the variances previously explained by the self-determination theory needs (see Appendix B). We thus find strong evidence that within everyday life interactions of recent migrants with majority outgroup members, the perception that one’s interaction-specific needs are fulfilled offers a meaningful and flexible predictor of interaction quality, outgroup attitudes, and well-being.

### Limitations

While we believe that a need fulfillment mechanism should be relevant to any intergroup contact, our samples focused on a minority- and (voluntary) migrant perspective. Without additional evidence, it thus remains difficult to judge whether motivational effects will generalize to other migrant groups (e.g., forced migrants), other intergroup contexts (e.g., gender-, religious-, or sexual orientation groups), or to majority groups and their outgroup attitudes. We sought to replicate our results in three studies with different types of migrants but the fact remains that all three studies had slightly more women than men participating and were younger and more educated samples overall. While the samples were representative of the migrant group to the focus region, the generalizability of the sample is restricted by its characteristics. We know of no research suggesting that in other contexts need fulfillment would be less relevant but future research may extend our findings to build an even broader understanding of need fulfillment in intergroup contacts. Researchers may even seek to explore the role of need fulfillment in real-world interactions more broadly (also see Downie et al., 2008).

A second limitation lies in our methodology. While intensive longitudinal data is close to real-life events, this method comes at the expense of longer and more robust scales. Long and repetitive scales are often not feasible in intensive longitudinal methods because of the increased burden to the participants. To circumvent this shortcoming, we have ensured that the measures we used were, whenever possible, based on past validations. However, the circumstance remains that intensive longitudinal data often does not allow the same scrutiny of measurement reliability as single-shot cross-sectional data sets. An additional methodological question lies in the unexplored potential of the longitudinal aspects of our data. For our research questions, we have focused on contemporaneous effects within the data set, yet future investigations should seek to extend the mechanism to developmental trajectories within and between participants.

A third limitation lies in the outcome variable we chose. We have focused on outgroup attitudes to ensure comparability to the past literature on the contact hypothesis and to replicate the most reliable patterns within ESM data (Pettigrew & Tropp, 2006). However, especially for disadvantaged minority members positive outgroup attitudes may entail negative downstream consequences such as a reduced endorsement of social change—even in the face of injustice (e.g., Dixon et al., 2012). We have additionally tested our need-fulfillment mechanism for well-being reports to test the direct benefits of the mechanism to lived realities. However, we are among the first to collect intensive longitudinal data on the experience of minority migrants and it remains an open empirical question whether the more positive outgroup attitudes following need-fulfilling interactions might ironically exacerbate inequalities for the disadvantaged group (also see Reimer & Sengupta, 2023).

Finally, our conceptualization of situational needs has been focused on the most essential test of a motivational mechanism. This comes at the expense of specificity in the situational motives (i.e., we have not explored whether different individual motives have stronger effects on interaction quality and outgroup attitudes). Such an investigation would be possible with the adaptive measurement we used (e.g., by looking at the differential effects of the clustered motives) but would not have been relevant to our theory–focused research question. Identifying cases where a specific minority faces a common need frustration could be instrumental in addressing systemic challenges. Future research may, thus, explore which situations activate or threaten specific motives (e.g., Gollwitzer & Wicklund, 1985; Leander et al., 2020) and which exact motives are most important in different intergroup contexts.

### Implications

Despite these limitations, we can nonetheless draw a number of implications for other researchers and practitioners—ranging from the benefits of longitudinal data to theoretical implications. The first implication concerns the feasibility and usefulness of intensive longitudinal data for intergroup contact research and the broader field of social psychology. While setting up an intensive longitudinal study is not easy, we believe the efforts to be similar to a sizable cross-sectional data collection (i.e., for a longitudinal or a high-quality cross-sectional data set with more than 3,000 intergroup interactions captured and more than 10,000 data points in total). Intensive longitudinal data, thus, opens up the possibility to explore research questions that focus on real-life phenomena outside the lab or focus on phenomena that depend on changes and influences over time. In the context of intergroup contact research, we are among the first to answer calls to test intergroup contact mechanisms using extended real-life data (e.g., MacInnis & Page-Gould, 2015; Pettigrew & Tropp, 2011). In doing so, we not only collected an unprecedented amount of real-life data, but our consideration of intensive longitudinal data may present new inconsistencies in how participants perceive and cognitively aggregate their past interactions with other groups—which may suggest large-scale recall biases or conflations of within- and between-participant effects in conventional cross-section studies.

A broader theoretical implication relates to the role of situational motivation in intergroup contacts. Our results offer the first promising test of a psychological mechanism of need fulfillment in intergroup contact. While our results are tentative given their novelty within the field, they were highly consistent across studies and may offer new theoretical avenues. Experiences of need fulfillment are a facet of the human experience that has thus far been underemphasized in the intergroup contact literature. This stands in stark contrast to the many cognitive (e.g., Brown & Hewstone, 2005; Pettigrew, 1998) and emotional aspects investigated within the field (e.g., Stephan et al., 2008). Future research may, therefore, be able to integrate broader theoretical frameworks of intergroup contact (e.g., motivations guiding cognition and affect, which in turn drive behavior. cf., theory of reasoned goal pursuit; Ajzen & Kruglanski, 2019. Also see Kreienkamp et al., 2023).

In addition, situational motivations in intergroup contact also offer promising avenues for practitioners and policy-makers. Intergroup contact theory is among the most implemented psychological theories (e.g., Al Ramiah & Hewstone, 2012; Pettigrew & Tropp, 2006; Reimer et al., 2021). Given our findings that need fulfillment in everyday intergroup contacts was at least as powerful as Allport’s conditions in predicting outgroup attitudes, considerations of people’s needs offer a substantially more immediate mechanism to address. In cases where some or all optimal contact conditions are not possible to be fulfilled, needs offer an even more compelling alternative (e.g., where equal status is contextually not possible or in cases where people help despite a lack of institutional support). In addition, our conceptualization of situational needs offers a clear opportunity for practitioners and interventions. Instead of addressing needs as a one-size-fits-all solution (e.g., simply focusing on competence needs), one may at times ask outgroup interaction partners what they need during an interaction. This is not to say that we should not explore which motives tend to be relevant to specific groups in specific intergroup contact contexts. Rather, during interventions for which data on important need contents are not available or infeasible to collect, a flexible and reactive approach of inquiring momentary intergroup contact needs might be more fruitful.

## Conclusion

In sum, we used intensive longitudinal methodologies to capture real-life interactions of recent migrants with the majority outgroup. Our three studies showcase the feasibility and utility of such data to test intergroup contact theory. We provide evidence that the fulfillment of situational needs during real-life intergroup contacts meaningfully predicts perceived interaction quality and positive outgroup attitudes. Our results point to motivational needs as an understudied aspect of intergroup contact that is important in understanding when and why an interaction is perceived as positive and will lead to more positive outgroup attitudes.

## Supplemental Material

sj-docx-1-psp-10.1177_01461672231204063 – Supplemental material for Need Fulfillment During Intergroup Contact: Three Experience Sampling Studies Supplemental material, sj-docx-1-psp-10.1177_01461672231204063 for Need Fulfillment During Intergroup Contact: Three Experience Sampling Studies by Jannis Kreienkamp, Maximilian Agostini, Laura F. Bringmann, Peter de Jonge and Kai Epstude in Personality and Social Psychology Bulletin

sj-docx-2-psp-10.1177_01461672231204063 – Supplemental material for Need Fulfillment During Intergroup Contact: Three Experience Sampling Studies Supplemental material, sj-docx-2-psp-10.1177_01461672231204063 for Need Fulfillment During Intergroup Contact: Three Experience Sampling Studies by Jannis Kreienkamp, Maximilian Agostini, Laura F. Bringmann, Peter de Jonge and Kai Epstude in Personality and Social Psychology Bulletin

sj-docx-3-psp-10.1177_01461672231204063 – Supplemental material for Need Fulfillment During Intergroup Contact: Three Experience Sampling Studies Supplemental material, sj-docx-3-psp-10.1177_01461672231204063 for Need Fulfillment During Intergroup Contact: Three Experience Sampling Studies by Jannis Kreienkamp, Maximilian Agostini, Laura F. Bringmann, Peter de Jonge and Kai Epstude in Personality and Social Psychology Bulletin

sj-docx-4-psp-10.1177_01461672231204063 – Supplemental material for Need Fulfillment During Intergroup Contact: Three Experience Sampling Studies Supplemental material, sj-docx-4-psp-10.1177_01461672231204063 for Need Fulfillment During Intergroup Contact: Three Experience Sampling Studies by Jannis Kreienkamp, Maximilian Agostini, Laura F. Bringmann, Peter de Jonge and Kai Epstude in Personality and Social Psychology Bulletin

## Appendix A

### Hypotheses and Analysis Plan

In this appendix, we present the expanded hypotheses and their associated analysis plan. Given the nested structure of much of our data, we test many of our hypotheses using a multilevel approach, where *y_ti_* denotes the response at measurement occasion *t* (*t =* 1,. . .,*T_i_*; level 1) for individual *i* (*i =* 1,. . .,*n*; level 2). All multilevel assumptions are tested as follows (e.g., for random slopes model with *j* within-person predictors):

(1)
$$\mathrm{Level}\;1\;\mathrm{Variance}:e_{ti}∼N\left(0,\sigma^{2}\right)$$

(2)
$$\mathrm{Level}\;2\;\mathrm{Variance}:\left[\begin{array}{c} u_{0,i}\\ \vdots \\ u_{j,i} \end{array}\right]\sim N\left(\begin{array}{cc} \left[\begin{array}{c} 0\\ \vdots \\ 0 \end{array}\right], & \left[\begin{array}{ccc} \tau_{00}^{2} & & \\ \vdots & \ddots & \\ \tau_{j0}^{2} & \cdots & \tau_{jj}^{2} \end{array}\right] \end{array}\right)$$

For our main aims we sequentially focus on four sets of models to test and validate our hypotheses:

1. Contact hypothesis in intensive longitudinal data.

Within the individual studies, we begin by testing the most basic assumption of the intergroup contact hypothesis that outgroup attitudes should be more positive after outgroup interactions but not after non-outgroup interactions. For this we use multilevel regression analyses, predicting outgroup attitudes from outgroup interaction and non-outgroup interaction dummy variables. We also include the participant means as level two predictors to fully disentangle within- (Level 1) and between-participant (Level 2) effects of intergroup contact (e.g., Snijders & Bosker, 2012, Section 4.6).

**Hypothesis 1 (H1):** Based on the most general understanding of the contact hypothesis, positive intergroup contacts should be associated with more favorable outgroup attitudes across intensive longitudinal data.
**Hypothesis 1a (H1a):** Outgroup attitudes should be more positive after an intergroup interaction compared to a non-outgroup interaction.

(3)
$$\begin{array}{rr} Level\;1: & \begin{array}{rr} Attitude_{ti}= & \beta_{0i}+\beta_{1i}OutgroupInteraction_{ti}+\\ & \beta_{2i}NonOutgroupInteraction_{ti}+e_{ti} \end{array}\\ Level\;2: & \begin{array}{rr} \beta_{0i}= & \gamma_{00}+\gamma_{01}MeanOutgroupInteraction_{i}+\\ & \gamma_{02}MeanNonOutgroupInteraction_{i}+u_{0i}\\ \beta_{1i}= & \gamma_{10}+u_{1i}\\ \beta_{2i}= & \gamma_{20}+u_{2i} \end{array} \end{array}$$

We then seek to test the full contact hypothesis by investigating intergroup contacts and perceived interaction quality jointly. To do so, we conduct a linear regression using person-level aggregated data from all three studies. In particular, we aggregate the number of outgroup interactions participants had, their average interaction quality perceptions, as well as their average outgroup attitudes. This approach has three main benefits: (a) Interaction quality ratings are only available if participants had an interaction, and the aggregation deals with this structural missingness. (b) Using the participant-level data from all three studies, we avoid potential power issues. (c) This analysis mimics the analyses conducted within the cross-section literature, where participants are asked to recall how many interactions they had over a one-month period, how positive these interactions were, and what their general attitudes toward the outgroup are.

**Hypothesis 1b (H1b):** Participants with more intergroup interactions should have more favorable outgroup attitudes.

(4)
$$r_{\left(ContactFrequency_{i},\;AverageQuality_{i}\right)}\neq 0$$

**Hypothesis 1c (H1c):** Participants with more intergroup interactions should have more favorable outgroup attitudes depending on the average interaction quality.

(5)
$$\begin{array}{rr} AverageAttitude_{i}= & \beta_{0}+\beta_{1}ContactFrequency_{i}+\\ & \beta_{2}AverageQuality_{i}+\\ & \beta_{3}ContactFrequency_{i}*AverageQuality_{i} \end{array}$$

We additionally control for the participant’s study membership.

Because this analysis uses the data from all three studies, the results of this analysis are presented in the “Robustness, Stability, and Embeddedness across Studies” section.

2. Situational need fulfillment during intergroup contact.

The main proposal of this manuscript has been the assertion that the fulfillment of situational needs during interaction will be associated with more positive interaction quality perceptions and, ultimately, more positive outgroup attitudes. Thus, for the main set of analyses, we focus on the reported outgroup interactions only. For each study, we will use multilevel regressions to test the main three assertions of our proposal (mirroring the basic steps of a traditional mediation analysis).

**Hypothesis 2 (H2):** Based on our proposal, intergroup interactions with higher situational need fulfillment should predict more favorable outgroup attitudes due to more positive interaction quality perceptions.
**Hypothesis 2a (H2a):** Situational need fulfillment during outgroup interactions should predict more positive outgroup attitudes.

(6)
$$\begin{array}{rr} Level\;1: & Attitude_{ti}=\beta_{0i}+\beta_{1i}SitNeedFulfill_{ti}+e_{ti}\\ Level\;2: & \beta_{0i}=\gamma_{00}+u_{0i}\\ & \beta_{1i}=\gamma_{10}+u_{1i} \end{array}$$

**Hypothesis 2b (H2b):** Situational need fulfillment during outgroup interactions should also predict higher perceived interaction quality.

(7)
$$\begin{array}{rr} Level\;1: & InteractionQuality_{ti}=\beta_{0i}+\beta_{1i}SitNeedFulfill_{ti}+e_{ti}\\ Level\;2: & \beta_{0i}=\gamma_{00}+u_{0i}\\ & \beta_{1i}=\gamma_{10}+u_{1i} \end{array}$$

**Hypothesis 2c (H2c):** The effect of situational need fulfillment on outgroup attitudes should be reduced when considered together with perceived interaction quality.

(8)
$$\begin{array}{rr} Level\;1: & \begin{array}{rr} Attitude_{ti}= & \beta_{0i}+\beta_{1i}SitNeedFulfill_{ti}+\\ & \beta_{2i}InteractionQuality_{ti}+e_{ti} \end{array}\\ Level\;2: & \beta_{0i}=\gamma_{00}+u_{0i}\\ & \beta_{1i}=\gamma_{10}+u_{1i}\\ & \beta_{2i}=\gamma_{20}+u_{2i} \end{array}$$

3. Allport’s conditions in intensive longitudinal data.

Within the third study, we formally measure all of Allport’s optimal contact conditions. We use multilevel regression models to test whether the fulfillment of Allport’s conditions in real-life data predicts more positive outgroup attitudes and higher perceived interaction quality, using the same approach as for the situational need fulfillment above.

**Hypothesis 3 (H3):** Based on Allport’s optimal contact conditions, intergroup interactions with equal status, common goals, collaboration, and structural support should predict more favorable outgroup attitudes due to more positive interaction quality perceptions.
**Hypothesis 3a (H3a):** Higher fulfillment of Allport’s conditions during outgroup interactions should predict more positive out-group attitudes.

(9)
$$\begin{array}{rr} Level\;1: & Attitude_{ti}=\beta_{0i}+\beta_{1i}Allport_{ti}+e_{ti}\\ Level\;2: & \beta_{0i}=\gamma_{00}+u_{0i}\\ & \beta_{1i}=\gamma_{10}+u_{1i} \end{array}$$

**Hypothesis 3b (H3b):** Higher fulfillment of Allport’s conditions during out-group interactions should also predict higher perceived interaction quality.

(10)
$$\begin{array}{rr} Level\;1: & InteractionQuality_{ti}=\beta_{0i}+\beta_{1i}Allport_{ti}+e_{ti}\\ Level\;2: & \beta_{0i}=\gamma_{00}+u_{0i}\\ & \beta_{1i}=\gamma_{10}+u_{1i} \end{array}$$

**Hypothesis 3c (H3c):** The effect of higher fulfillment of Allport’s conditions on outgroup attitudes should be reduced when considered together with perceived interaction quality.

(11)
$$\begin{array}{rr} Level\;1: & \begin{array}{rr} Attitude_{ti}= & \beta_{0i}+\beta_{1i}Allport_{ti}+\\ & \beta_{2i}InteractionQuality_{ti}+e_{ti} \end{array}\\ Level\;2: & \beta_{0i}=\gamma_{00}+u_{0i}\\ & \beta_{1i}=\gamma_{10}+u_{1i}\\ & \beta_{2i}=\gamma_{20}+u_{2i} \end{array}$$

We then compare the effect of Allport’s contact conditions with our situational need fulfillment by comparing the model fit statistics of the two individual models and by adding both concepts to a joint multilevel regression model to see whether the two approaches explain the same variance in out-group attitudes.

**Hypothesis 4 (H4):** Based on our proposal, the fulfillment of the situational need should predict outgroup attitudes at least as well as Allport’s conditions.
**Hypothesis 4a (H4a):** The need model (H2a) should predict more variance in outgroup attitudes than the model based on Allport’s conditions (H3a).

(12)
$$\begin{array}{rr} AIC_{KeyNeedModel} & <AIC_{AllportModel}\\ BIC_{KeyNeedModel} & <BIC_{AllportModel} \end{array}$$

**Hypothesis 4b (H4b):** The effect of situational need fulfillment on outgroup attitudes should persist even when taking other Allport’s conditions into account. Thus, the effect of situational need fulfillment on outgroup attitudes should remain strong even after controlling for Allport’s conditions.

(13)
$$\begin{array}{rr} Level\;1: & \begin{array}{rr} Attitude_{ti}= & \beta_{0i}+\beta_{1i}SitNeedFulfill_{ti}+\\ & \beta_{2i}Allport_{ti}+e_{ti} \end{array}\\ Level\;2: & \beta_{0i}=\gamma_{00}+u_{0i}\\ & \beta_{1i}=\gamma_{10}+u_{1i}\\ & \beta_{2i}=\gamma_{20}+u_{2i} \end{array}$$

4. Robustness, stability, and embeddedness across studies

Within the final set of analyses, we look at the broader picture of our results and leverage the data from all participants to contextualize our results.

### Robustness Within Studies

To build further confidence in the effect of situational need fulfillment during out-group interactions, we conducted two additional robustness analyses for each study.

First, to check for the role of alternate psychological needs, we add the fulfillment of self-determination theory needs (i.e., competence, autonomy, and relatedness) to the multilevel regression. We then also compare the model with models that predicts outgroup attitudes from self-determination theory need fulfillments or situational need fulfillments only.

**Hypothesis 5 (H5):** The effect of situational need fulfillment on outgroup attitudes should persist even when taking other fundamental psychological needs into account. Thus, the effect of situational need fulfillment on out-group attitudes should remain strong even after controlling for autonomy, competence, and relatedness fulfillment during the interaction (cf., self-determination theory

(14)
$$\begin{array}{rr} Level\;1: & \begin{array}{rr} Attitude_{ti}= & \beta_{0i}+\beta_{1i}SitNeedFulfill_{ti}+\beta_{2i}Autonomy_{ti}+\\ & \beta_{3i}Competence_{ti}+\beta_{4i}Relatedness_{ti}+e_{ti} \end{array}\\ Level\;2: & \beta_{0i}=\gamma_{00}+u_{0i}\\ & \beta_{1i}=\gamma_{10}+u_{1i}\\ & \beta_{2i}=\gamma_{20}+u_{2i}\\ & \beta_{3i}=\gamma_{30}+u_{3i}\\ & \beta_{4i}=\gamma_{40}+u_{4i} \end{array}$$

To ensure that the situational need fulfillment is outgroup contact specific, we return to the full sample of intensive longitudinal measurements within each study and test whether there is an interaction effect of out-group contact (vs. no out-group contact) and situational need fulfillment. We expect that the effect of situational need fulfillment is specific to outgroup interactions and not merely due to a more need-fulfilled life in general.

**Hypothesis 6 (H6):** The effect of situational need fulfillment on outgroup attitudes should be specific to intergroup interactions and not be due to need fulfillment in general. Thus, the effect of situational need fulfillment on out-group attitudes should be stronger for intergroup interactions than for ingroup interactions.

(15)
$$\begin{array}{rr} Level\;1: & \begin{array}{rr} Attitude_{ti}= & \beta_{0i}+\beta_{1i}SitNeedFulfill_{ti}+\\ & \beta_{2i}OutgroupInteraction_{ti}+\\ & \beta_{3i}SitNeedFulfill*OutgroupInteraction_{ti}+e_{ti} \end{array}\\ Level\;2: & \beta_{0i}=\gamma_{00}+u_{0i}\\ & \beta_{1i}=\gamma_{10}+u_{1i}\\ & \beta_{2i}=\gamma_{20}+u_{2i}\\ & \beta_{3i}=\gamma_{30}+u_{3i} \end{array}$$

The results of the robustness analyses are presented in Appendix B to allow for a more concise presentation of our main hypotheses in the main text.

### Stability Across Studies

We assess the stability of our main analyses. We use forest plots (including meta-analytical estimates) to visualize the direction and effect sizes of our three studies.

**Hypothesis 7 (H7):** *The effects of our main hypotheses and robustness analyses should be consistent across studies.*

### Embeddedness of Code Needs

We, finally, use the qualitative data from the participants’ self-identified situational needs to contextualize our results. We leverage machine learning to extract a topic model of the free-text entries across the three studies. We describe the extracted topics and themes and compare them to the needed contents usually found and measured within the psychological literature. Full methodological details and visualizations are available in Supplemental Material C.

*This analysis is data-driven and exploratory. As such, the analysis has no associated hypothesis*

## Appendix B

### Robustness Analyses

In this appendix, we present the empirical details of our additional robustness analyses. These analyses are specifically designed to check for alternative models and contextualize our results. We (a) check whether situational need fulfillment is indeed outgroup contact specific. For this analysis, we return to the full sample of intensive longitudinal measurements and test whether there is an interaction effect of outgroup contact (vs. no outgroup contact) and situational need fulfillment. We expect that the effect of situational need fulfillment is specific to outgroup interactions and not merely due to a more need-fulfilled life in general. We then check (b) whether the need mechanism is relevant to both planned and accidental outgroup interactions, and (c) extends to the more individual-focused experience of well-being. In a final set of analyses, we (d) check whether the need fulfillment mechanism is relevant to different types of need content (i.e., motives) and (e) remains relevant even when accounting for the fulfillment of fundamental psychological needs (i.e., self-determination theory needs: competence, autonomy, and relatedness).

As with the main analyses, full surveys are available in our OSF repository (Kreienkamp et al., 2022b) and the full data description is available in Online Supplementary Material A. Correlations and descriptive statistics of the included variables are available in Tables 1 and 2.

### Additional Materials

In addition to the measurement of whether or not participants had an intergroup interaction and their situational need fulfillment, we also included a number of variables that allowed us to assess the robustness of our results.

**Table B1.** Full Sample: Correlation Table and Descriptive Statistics.

Variable	Correlations							Descriptives				
	Sit. Need	Competence	Autonomy	Relatednesss	Accidental	Quality	Attitudes NL	Grand Mean	Between *SD*	Within *SD*	ICC(1)	ICC(2)
Study 1												
Sit, Need		.36***	.31***	.05			0.11***	77.95	14.68	20.83	0.29	0.96
Competence	.68***		.45***	.09**			0.14***	62.10	13.72	20.89	0.28	0.95
Autonomy	.54***	.72***		.09**			0.12***	72.17	12.09	15.15	0.38	0.97
Relatednesss	.01	−.14	−.05				0.14***	55.29	14.59	23.29	0.28	0.95
Attitudes NL	−.23	−.20	.19	−.06				71.49	12.91	8.11	0.70	0.99
Study 2												
Sit. Need		.24***	.15***	.17***	−.11***	.22***	0.08***	84.87	9.17	20.33	0.15	0.89
Competence	.55***		.45***	.35***	−.05*	.36***	0.11***	72.55	14.47	21.17	0.30	0.95
Autonomy	.62***	.69***		.34***	−.11***	.42***	0.05**	82.59	11.21	16.06	0.32	0.95
Relatednesss	.34***	.62***	.52***		−.09***	.52***	0.10***	61.21	13.36	28.74	0.17	0.90
Accidental	−.49***	−.22*	−.32***	−.07		−.24***	0.03	25.09	14.62	29.13	0.17	0.85
Quality	.54***	.68***	.59***	.61***	−.33***		0.09***	74.51	11.24	16.59	0.29	0.92
Attitudes NL	.12	.07	−.07	−.12	.02	.13		67.26	18.64	9.40	0.80	0.99
Study 3												
Sit. Need		.27***	.31***	.20***	−.10***	.37***	0.10***	83.57	8.02	17.14	0.18	0.92
Competence	.48***		.55***	.39***	−.14***	.38***	0.06**	77.45	11.49	18.92	0.26	0.95
Autonomy	.57***	.78***		.51***	−.16***	.54***	0.01	83.76	9.72	15.87	0.28	0.96
Relatednesss	.22	.51***	.48***		−.13***	.50***	−0.04*	63.44	13.34	28.85	0.17	0.92
Accidental	−.50***	−.24*	−.27*	−.04		−.21***	0.02	24.73	14.98	28.51	0.21	0.92
Quality	.39***	.54***	.59***	.53***	−.02		0.08***	76.62	12.42	16.98	0.34	0.96
Attitudes NL	.11	.12	.11	.20	.02	.11		64.77	14.37	10.88	0.66	0.99
Across Studies												
Sit. Need		.31***	.28***	.22***	−.14***	.32***	0.14***	83.81	7.85	20.24	0.11	0.92
Competence	.49***		.55***	.41***	−.11***	.43***	0.11***	73.38	11.52	22.25	0.18	0.95
Autonomy	.56***	.71***		.44***	−.14***	.51***	0.09***	82.36	9.35	16.98	0.21	0.96
Relatednesss	.25*	.56***	.50***		−.12***	.52***	0.08***	61.55	10.73	29.45	0.11	0.91
Accidental	−.47***	−.23*	−.34***	−.01		−.22***	−0.01	24.56	12.38	29.70	0.12	0.89
Quality	.39***	.57***	.54***	.57***	−.14		0.17***	75.46	9.44	17.85	0.19	0.93
Attitudes NL	−.04	.04	−.06	−.14	.11	.00		66.58	14.46	13.25	0.49	0.99

*Note.* Interaction Intent and Interaction Quality were only measured for out-group interactions in Study 1. Sit. = situational, Attitudes NL = Attitudes towards the Dutch, ICC = Intraclass Correlation Coefficient; Upper triangle: Within-person correlations; Lower triangle: Between-person correlations.

* *p* < .05. ***p* < .01. ****p* < .001.

**Table B2.** Out-group Interaction Sample: Correlation Table and Descriptive Statistics.

Variable	Correlations							Descriptives				
	Sit. Need	Competence	Autonomy	Relatedness	Accidental	Quality	Attitudes NL	Grand Mean	Between *SD*	Within *SD*	ICC(1)	ICC(2)
Study 1												
Sit. Need		.33***	.26***	.19***	.03	0.35***	0.28***	81.91	12.67	17.86	0.33	0.89
Competence	.84***		.50***	.27***	.06	0.41***	0.26***	65.96	13.49	18.88	0.31	0.88
Autonomy	.59***	.86***		.24***	.01	0.36***	0.27***	72.00	10.02	15.12	0.28	0.86
Relatednesss	.27	.16	.22		.19***	0.53***	0.40***	51.41	14.50	23.85	0.23	0.84
Accidental	−.01	.14	.05	−.15		−0.05	0.11	28.41	21.39	26.79	0.36	0.91
Quality	.41	.23	.21	.36	.01		0.57***	65.89	7.94	18.51	0.13	0.72
Attitudes NL	−.02	−.26	.05	.20	.21	0.38		72.56	13.97	9.73	0.66	0.97
Study 2												
Sit. Need		.23***	.12***	.18***	−.12***	0.22***	0.16***	86.86	11.20	15.87	0.14	0.58
Competence	.45**		.48***	.32***	−.03	0.38***	0.19***	73.23	13.95	16.81	0.27	0.76
Autonomy	.48***	.60***		.24***	.03	0.36***	0.15***	78.58	14.07	14.24	0.40	0.85
Relatednesss	−.21	.41**	.24		.10**	0.46***	0.22***	60.30	17.35	26.14	0.19	0.67
Accidental	−.60***	−.18	−.27*	.21		−0.16***	−0.01	33.86	23.37	29.88	0.28	0.77
Quality	.26	.47***	.52***	.50***	−.24		0.29***	67.08	12.54	16.54	0.24	0.73
Attitudes NL	.18	.24	.09	−.10	−.10	0.09		70.41	17.13	9.87	0.72	0.96
Study 3												
Sit. Need		.23***	.26***	.15***	−.05*	0.35***	0.24***	84.84	9.27	13.00	0.30	0.91
Competence	.53***		.52***	.33***	−.09***	0.31***	0.20***	75.94	12.23	17.21	0.29	0.91
Autonomy	.57***	.79***		.43***	−.07**	0.46***	0.20***	79.07	12.88	15.26	0.36	0.93
Relatednesss	.12	.40***	.40***		.00	0.43***	0.20***	59.62	19.26	23.45	0.34	0.93
Accidental	−.46***	−.27*	−.29*	.04		−0.16***	−0.05*	29.13	17.94	30.37	0.26	0.89
Quality	.48***	.58***	.62***	.47***	−.06		0.29***	71.95	14.97	16.71	0.43	0.95
Attitudes NL	.24	.32**	.31**	.35**	−.12	0.32**		68.24	13.72	11.23	0.63	0.98
Across Studies												
Sit. Need		.28***	.24***	.16***	−.12***	0.30***	0.21***	85.92	9.31	15.80	0.17	0.85
Competence	.46***		.53***	.33***	−.07***	0.38***	0.18***	73.91	11.29	18.17	0.19	0.87
Autonomy	.55***	.69***		.36***	−.05**	0.44***	0.20***	79.05	11.31	15.33	0.28	0.91
Relatednesss	.01	.34**	.26*		.03	0.45***	0.22***	59.81	15.05	26.21	0.18	0.85
Accidental	−.40***	−.21	−.36***	.10		−0.15***	−0.04*	29.66	18.06	30.09	0.17	0.85
Quality	.36***	.45***	.50***	.41***	−.13		0.31***	69.78	11.31	17.77	0.24	0.89
Attitudes NL	.06	.24*	.07	.02	.03	0.05		69.69	13.63	12.67	0.43	0.95

*Note.* Sit. = situational, Attitudes NL = Attitudes towards the Dutch, ICC = Intraclass Correlation Coefficient; Upper triangle: Within-person correlations; Lower triangle: Between-person correlations.

* *p* < .05. ***p* < .01. ****p* < .001.

#### Specific Psychological Needs

. In addition to the intergroup contact dummy and situational need reported in the main text, we included a common measure of three self-determination theory needs (see Downie et al., 2008). The measurement was identical in all three studies. The items were introduced either by “*During the interaction”*: or “*This morning [/afternoon]”*: and measured autonomy (“*I was myself.*”), competence (“*I felt competent.*”), and relatedness (without intergroup contact “*I had a strong need to belong*”; with intergroup contact: “*I shared information about myself.*” and “*The other(s) shared information about themselves.*”). All items were rated on a continuous slider scale from very little (−50) to a great deal (+50).

#### Interaction Intent

To assess whether an interaction was accidental (vs. planned), we asked participants with a single item to report the extent to which “The interaction with -X- was accidental.” The respondents were asked to report this context variable for all interactions they reported on using a continuous slider ranging from “not at all” (0), through “very little” (33) and “somewhat” (66), to “a great deal” (100). In all studies the scale showed a right skew (*M* = 29.60, *SD* = 33.68).

#### Goal-Directedness

To assess whether the need content (i.e., the motives) would impact the effect of the need fulfillment experiences, we manually coded the topics we extracted during the topic modeling on two dimensions of how much they reflect a practical and a psychological goal-directedness. We chose practical and psychological needs specifically as our dimensions to account for differences in the types of needs that participants commonly reported. With practical motives, we refer to specific, tangible goals or tasks that participants aimed to accomplish during the interaction. These instrumental goals are usually observable, concrete, and often centered on external outcomes, such as acquiring resources, completing tasks, and addressing immediate challenges (e.g., Oduntan & Ruthven, 2019). With psychological motives, we refer to underlying motives or desires that are more abstract and relate to personal fulfillment and well-being. In contrast to practical needs, psychological needs delve into the subjective and internal aspects of human experiences. These needs pertain to emotions, social connections, and cognitive processes, reflecting individuals’ quest for personal growth, well-being, and thriving in social relationships (Dweck, 2017). Note that with this approach any particular motive can include a practical and/or psychological goal-directedness but can also be classified as not having any goal at all. The full coding protocol we developed with examples for each of the codes is available in our Online Supplemental Material D.

After initial training, each of the two coders independently coded the 47 topics on the two dimensions, using one of three options each (i.e., 0 = *no goal*, 1 = *vague goal*, 2 = *clear goal*). Interrater reliability assessments showed that for both the practical and the psychological needs, agreement was not optimal using the three answer options (agreement practical = 72%, agreement psychological = 78%). However, most disagreements were, if a need was present, whether that need was *vague* (1) or *concrete* (2). We, thus, collapsed these two categories making the ratings binary (need absent vs. need present). With the simpler coding, interrater agreement for practical needs (93%) and the psychological need (96%) were much more reliable. Using Cohen’s *κ* as our measure of inter-rater reliability, we find that both the practical need coding (*Cohen’s κ* = 0.80, 95% CI: [0.58, 1.00]) as well as the psychological need coding (*Cohen’s κ* = 0.86, 95% CI: [0.67, 1.00]) were very good. We thus proceeded with this collapsed coding. After resolving coder disagreements and merging the codings back to the free-text responses, we found that a majority of responses showed both a practical and a psychological need (57.95%) and only few responses had no goal at all (1.03%) with the remaining 41.02% having either a practical or a psychological need only (see Online Supplemental Material A for more detailed tables and visualizations).

#### Well-Being

We measured experienced well-being using a visual analog scale adapted from Davies et al. (2022). Participants were asked to respond to the question “How do you feel right now?” using a continuous visual slider ranging from “very sad”(−100) to “very happy” (100). The well-being ratings were generally normally distributed (*M* = 64.80, *sd* = 19.25).

### Results

To build further confidence in our results, we assessed a number of additional models that might offer alternative explanations. We will discuss the results in sequential order—in every case first considering the a global test of the model across the three studies and only then assessing whether the global three-level regression model suppresses any important person-level variations within the studies.

#### Contact Specific

. We begin our robustness analysis by testing whether the effect of situational need fulfillment is specific to an actual outgroup contact, rather than need fulfillment in general. For this, we analyzed the generalized situational need fulfillment (either during contact or about the daytime in general) and tested whether the effect differed during experience sampling measurements with and without outgroup contacts. We start this test by assessing the effect across all three studies, using a three-level hierarchical model, where measurements are nested within participants, and participants are nested within studies. In this overall model, we found no main effect of situational need fulfillment, random slopes model, grand-mean standardized to account for all levels of variance; *b* = 0.62, *t*(3.187) = 2.89, *p* = .058, 95% CI: [0.20, 1.03], but a significant interaction effect of situational need fulfillment and outgroup contact, *b* = 2.44, *t*(4,663.172) = 8.62, *p* < .001, 95% CI: [1.89, 3.00]; also see Table B3. While the three-level hierarchical model can be sensitive to scaling issues, this already indicates that it is not situational need fulfillment in general—but only situational need fulfillment during an outgroup contact that predicts more positive out-group attitudes.

To ensure that the results are not affected by scaling issues (e.g., study-level variances suppressing person-level variances) or a similar Simpson’s paradox, we additionally assess the model within each of the three studies. Within each of the three studies, the effects are more pronounced, so we also see a significant effect of situational need fulfillment (all *b* > 0.06, all *p* < .005) as well as outgroup contact itself (all|*b*| > 1.81, all *p* < .034) but the interaction effect consistently remains the most reliable predictor of outgroup attitudes (all|*b*| > .06, all *p* < .002, also see Table B3). There is thus consistent evidence that need fulfillment relates to outgroup attitudes for outgroup contacts in particular but not need fulfillment in general.

#### Interaction Intent

. Second, to assess whether the need fulfillment mechanism affected by whether the interaction was accidental or planned we ran an exploratory moderation analysis using the participants’ ratings of how much they perceived the interaction as “accidental.” It should be noted that we asked our participants to focus on most the important interaction (i.e., “*The following questions will be about the interaction you consider most significant.*”; emphasis as in original). We again start our analysis approach by assessing the model across all three studies, using a three-level hierarchical model. In this overall model, we retain the main effect of situational need fulfillment, random slopes model, grand-mean standardized to account for all levels of variance; *b* = 2.87, *t*(7.979) = 7.32, *p* < .001, 95% CI: [2.10, 3.64], but neither contact intent nor the moderation effect affect the results (all|*b*| < .27 and all *p* > .225; see Table B3 for full results).

We again sought to ensure that the results were not affected by scaling issues by additionally assessing the interaction intentionality model within each of the three studies. Within each of the three studies, the effect of situational need fulfillment became even clearer (all|*b*| > .13, all *p* < .006). But in none of the studies neither outgroup contact intention nor the moderation effect explained a significant amount of variance in outgroup attitudes (all|*b*| < .03 and all *p* > .073; also see Table B3). There is thus consistent evidence that need fulfillment is related to outgroup attitudes, even when taking the intentionality of the interaction into account—at least in our three samples and with a focus on the most significant interactions.

#### Well-Being Outcome

Third, to build a stronger case for the relevance of need fulfillment to minority group members, we exploratorily assessed the effect of need-fulfilling out-group interactions on self-reported well-being. We, thus, re-ran our main analysis but substituted the outgroup attitudes outcome with situational well-being. As with the previous robustness analyses, we begin with a global three-level hierarchical model (across the three studies). We find that need fulfillment during outgroup contacts, indeed, has a similar effect on experienced well-being, random slopes model, grand-mean standardized to account for all levels of variance; *b* = 3.50, *t*(4.219) = 6.33, *p* = .003, 95% CI: [2.42, 4.58]. We found the same result when we assessed each of the three studies individually. In each of the studies situational need fulfillment during the outgroup interaction was related with higher well-being ratings by the participants (random slopes model, centered within participants; all|*b*| > .10, all *p* < .009). We, thus, find consistent and meaningful evidence that need-fulfilling outgroup interactions also relate to higher everyday well-being.

#### Need Types

Fourth, to assess the role of different types of motives reported by our participants, we added our coding of practical and psychological goal-directedness as additional predictors to our base model. We thus had situational need fulfillment predicting outgroup attitudes while also accounting for whether the reported motives were capturing practical and/or psychological motives. We again ran a global model, across the three studies first. We found that situational need fulfillment remains a core predictor of outgroup attitudes, random slopes model, grand-mean standardized to account for all levels of variance; *b* = 2.27, *t*(48.194) = 2.72, *p* = .009, 95% CI: [0.63, 3.90], even after accounting for different types of motives. None of the motive types nor the moderation effects reached statistical significance within the overall analysis (all|*b*| < 1.45 and all *p* > .073; see Table B3 for full results).

When looking at the individual studies, we again saw that the effect of situational need fulfillment remained the only clear effect (all|b| > 0.14, all *p* < .015). In addition, in none of the studies neither motive type dummies nor the moderation effect explained a significant amount of variance in outgroup attitudes (all|b| < 1.53 and all p > 0.144; also see Table B3). We, thus, find consistent evidence that need fulfillment is related to outgroup attitudes, even when taking the type of need into account — at least in our three samples.

#### Specific Psychological Needs

In a final step, we checked whether during the interaction the situational need remains a meaningful predictor even when taking other fundamental psychological needs into account. We again take a two-step approach, starting with a cross-study global three-level test and then assessing the effects within the individual studies. Within the overall model, we find that across the studies situational need fulfillment remained a strong predictor of outgroup attitudes, even after controlling for the three self-determination theory need, random slopes model, grand-mean standardized to account for all levels of variance; *b* = 1.88, *t*(2.702) = 4.75, *p* = .022, 95% CI [1.11, 2.66]. Within this overall analysis, none of the self-determination theory needs independently predicted outgroup attitudes to a statistically significant extent (all *p* > .093). However, some of the effect sizes were largely comparable to that of the situational need fulfillment (all|*b*| < 2.10, particularly that of relatedness fulfillment; see Table B3 for full results).

**Table B3.** Robustness Analyses.

	Overall		Study 1		Study 2		Study 3	
Variable	B	β	B	β	B	β	B	β
Contact Specific								
(Intercept)	65.76** [49.86, 72.04]		71.60**** [66.33, 76.86]		68.22**** [64.95, 71.49]		65.17**** [62.00, 68.34]	
Situational Need	0.62 [−13.92, 14.10]	0.01 [−0.44, 0.45]	0.09** [0.04, 0.14]	0.21 [0.13, 0.28]	0.06**** [0.04, 0.08]	0.13 [0.08, 0.17]	0.11**** [0.08, 0.14]	0.13 [0.09, 0.17]
Out-group Interaction	3.59 [−20.13, 7.99]	−0.60 [−1.74, 0.53]	1.81* [0.25, 3.37]	0.28 [0.05, 0.50]	2.88*** [1.35, 4.40]	0.23 [0.16, 0.31]	5.41**** [3.72, 7.10]	0.37 [0.27, 0.48]
Situational Need × Out-group Interaction	2.44**** [0.09, 0.14]	0.01 [0.01, 0.01]	0.13**** [0.07, 0.18]	0.32 [0.19, 0.45]	0.06** [0.02, 0.10]	0.14 [0.06, 0.22]	0.17**** [0.12, 0.21]	0.19 [0.12, 0.26]
$R_{marginal}^{2}/R_{conditional}^{2}$	.000 / 1.000		.017 / .727		.005 / .818		.037 / .712	
Interaction Intent								
(Intercept)	70.14**** [42.53, 65.97]		71.43**** [65.57, 77.29]		70.40**** [67.36, 73.45]		68.16**** [64.95, 71.36]	
Situational Need	2.87**** [−8.58, 8.95]	0.02 [−1.22, 1.25]	0.17** [0.06, 0.29]	0.42 [0.26, 0.58]	0.13*** [0.07, 0.20]	0.25 [0.12, 0.37]	0.19**** [0.12, 0.26]	0.24 [0.15, 0.32]
Interaction Accidental	0.12 [−9.82, 9.92]	0.00 [−1.07, 1.07]	0.03 [0.00, 0.06]	0.13 [0.02, 0.24]	0.01 [−0.02, 0.03]	−0.02 [−0.09, 0.05]	−0.01 [−0.04, 0.01]	−0.02 [−0.08, 0.03]
Situational Need × Interaction Accidental	−0.27 [0.00, 0.00]	0.00 [0.00, 0.00]	0.00 [0.00, 0.00]	0.00 [−0.11, 0.11]	0.00 [0.00, 0.00]	−0.01 [−0.08, 0.07]	0.00 [0.00, 0.00]	−0.04 [−0.09, 0.02]
$R_{marginal}^{2}/R_{conditional}^{2}$	.000 / 1.000		.135 / —		.015 / .728		.021 / .687	
Well-being Outcome								
(Intercept)	65.25*** [28.58, 63.85]		61.34**** [55.56, 67.12]		67.18**** [64.66, 69.70]		63.63**** [60.85, 66.41]	
Situational Need	3.50** [−16.18, 16.62]	0.01 [−1.33, 1.35]	0.25** [0.10, 0.40]	0.31 [0.17, 0.46]	0.10** [0.04, 0.16]	0.17 [0.11, 0.23]	0.20*** [0.11, 0.29]	0.17 [0.10, 0.25]
$R_{marginal}^{2}/R_{conditional}^{2}$	.000 / 1.000		.054 / .541		.008 / .348		.017 / .375	
Need Type								
(Intercept)	69.40**** [50.54, 64.07]		73.09**** [66.87, 79.31]		70.37**** [67.24, 73.51]		68.18**** [64.77, 71.60]	
Situational Need	2.27** [0.09, 0.21]	0.01 [−0.82, 0.84]	0.14* [0.03, 0.26]	0.29 [0.12, 0.46]	0.14** [0.06, 0.21]	0.15 [0.04, 0.26]	0.19*** [0.08, 0.29]	0.18 [0.08, 0.29]
practical need	0.01 [−4.82, 7.25]	−0.11 [−1.00, 0.78]	−0.04 [−2.76, 2.69]	−0.06 [−0.33, 0.20]	0.80 [−1.22, 2.82]	0.17 [0.01, 0.32]	−0.99 [−2.85, 0.86]	−0.02 [−0.16, 0.12]
psychological need	1.45 [−8.48, 4.45]	−0.13 [−1.11, 0.84]	1.53 [−3.66, 6.73]	0.08 [−0.28, 0.43]	1.50 [−0.98, 3.98]	0.13 [−0.03, 0.30]	1.25 [−0.42, 2.93]	0.10 [−0.04, 0.24]
Situational Need × practical need	−0.23 [−0.08, 0.05]	0.00 [0.00, 0.01]	0.01 [−0.13, 0.15]	−0.12 [−0.36, 0.12]	−0.02 [−0.15, 0.10]	−0.04 [−0.23, 0.14]	−0.05 [−0.18, 0.08]	0.03 [−0.11, 0.18]
Situational Need × psychological need	0.67 [−0.03, 0.11]	0.00 [0.00, 0.01]	−0.10 [−0.31, 0.11]	−0.02 [−0.33, 0.29]	0.04 [−0.07, 0.15]	0.04 [−0.13, 0.21]	0.04 [−0.11, 0.19]	−0.06 [−0.22, 0.10]
$R_{marginal}^{2}/R_{conditional}^{2}$	.078 / —		.046 / —		.017 / .760		.018 / .696	
Specific Psychological Needs								
(Intercept)	70.93** [41.14, 58.40]		71.54**** [65.79, 77.28]		70.75**** [67.59, 73.90]		68.68**** [65.53, 71.82]	
Situational Need	1.88* [−5.45, 5.68]	0.01 [−1.13, 1.15]	0.10* [0.02, 0.18]	0.27 [0.11, 0.43]	0.06** [0.02, 0.10]	0.14 [0.06, 0.22]	0.14*** [0.07, 0.21]	0.18 [0.09, 0.26]
Autonomy	0.78 [−8.61, 8.70]	0.01 [−1.15, 1.16]	0.05 [−0.12, 0.23]	0.12 [−0.02, 0.26]	0.04 [−0.01, 0.09]	0.05 [−0.03, 0.13]	0.04 [0.00, 0.08]	0.05 [−0.03, 0.12]
Competence	0.95 [−3.70, 3.78]	0.00 [−1.11, 1.11]	0.05 [−0.16, 0.26]	0.02 [−0.21, 0.25]	0.05* [0.01, 0.10]	0.06 [−0.03, 0.15]	0.06* [0.01, 0.10]	0.09 [0.02, 0.16]
Relatednesss	2.10 [−4.04, 4.19]	0.01 [−1.20, 1.22]	0.10*** [0.06, 0.14]	0.29 [0.17, 0.41]	0.06**** [0.03, 0.09]	0.16 [0.09, 0.24]	0.06*** [0.02, 0.09]	0.10 [0.02, 0.19]
$R_{marginal}^{2}/R_{conditional}^{2}$	.176 / —		.061 / .860		.025 / .747		.041 / .702	

* *p* < .05. ***p* < .01. ****p* < .001. *****p* < .0001.

When looking at the individual studies, we again saw that situational need fulfillment remained a consistent predictor of outgroup attitudes, even after accounting for the self-determination theory need (all|*b*| > .06, all *p* < .030). However, across all three studies the fulfillment of relatedness motives also emerged as a consistent predictor of outgroup attitudes (all|*b*| > .06, all *p* < .001). In addition, in the larger Studies 2 and 3 competence fulfillment was also related to more positive out-group attitudes, Study 2: *b* = 0.05, *t*(841.8) = 2.43, *p* = .015, 95% CI: [0.01, 0.10], Study 3: *b* = 0.06, *t*(30.20) = 2.62, *p* = .013, 95% CI: [0.01, 0.10]. None of the autonomy fulfillment effects reached statistical significance nor did the competence fulfillment during study 1 (see Table B3 for the full results). In short, find that across our samples, relatedness fulfillment (and to a smaller extend competence fulfillment) are instrumental in understanding when an out-group contact leads to more positive out-group attitudes. Importantly, even when considering these effects situational need fulfillment remains a strong and consistent predictor of out-group attitudes. In some cases, we even find that situational need fulfillment takes on some of the variance that would otherwise be explained by the self-determination theory needs (see Online Supplemental Material A).

### Conclusion

Across the wide variety of robustness analyses, we thus find that the experience of need fulfillment is a robust and flexible predictor of positive out-group attitudes even when accounting for a range of other and even alternate predictors. However, we also find that the situational need-fulfillment mechanism does not account for all need-related variance in out-group attitudes. Notably, the fulfillment of relatedness (and to some extent competence) needs explained additional variance in out-group attitudes. Nonetheless, the situational need fulfillment remained extremely reliably a core predictor of out-group attitudes.

## Notes

1. In addition, such experience-sampling data can be collected close to the intergroup interactions and would, thus, largely mitigate recall biases. Moreover, because data is nested within participants, experience-sampling data often allows capturing large amounts of high-quality data with relatively few participants (Shiffman et al., 2008).

2. Two participants completed only two days (among the others, participation was 93.70%). These two participants also reported no out-group contacts. These participants are removed from any analyses that focus on out-group contacts.

3. Interestingly, adding random slopes to this model did not explain additional variance. This is unusual and might indicate that the effect is very consistent across participants. However, the small number of participants, or other measurement issues provide an alternative explanation, which is why we offer a combined data set analysis as part of our stability analyses.

4. It should be noted that these topic models are not without limitations, especially because they depend on a small set of hyperparameters that determine the characteristics of the embedding, dimension reduction, clustering, and core term extraction. The authors have also inspected a major subset of the free-text responses manually and are under the impression that the topics described here accurately represent the broader content.
