# Supplementary material for: Need Fulfillment During Intergroup Contact: Three Experience Sampling Studies
Source: Pers Soc Psychol Bull. 2023 Nov 21;51(6):1047–77. doi: 10.1177/01461672231204063 (PMC12044218; doi:10.1177/01461672231204063)
Supplement: sj-docx-1-psp-10.1177_01461672231204063 – Supplemental material for Need Fulfillment During Intergroup Contact: Three Experience Sampling Studies [file sj-docx-1-psp-10.1177_01461672231204063.docx]

Supplementary Information for

Need Fulfillment During Intergroup Contact: Three Experience

Sampling Studies

**Supplemental Information A: Annotated Analyses**

*Jannis Kreienkamp, Maximilian Agostini, Laura F. Bringmann, Peter de Jonge, Kai Epstude*

Corresponding Author: Jannis Kreienkamp

E-mail: j.kreienkamp@rug.nl

Last updated: June 1, 2023

1

# Supplemental Information A: Annotated Analyses

This supplementary information documents the methodology and results of all three studies. As part of our open supplemental materials, we share the full RMarkdown file which offers an annotated version of our all analysis steps. This file offers a transparent and reproducible analysis code, which includes full author commentary. We rendered the RMarkdown as an interactive HTML file, which we host as part of our open GitHub repository. We recommend the rendered version for almost all readers (full R code is also available via the rendered version).

<https://janniscodes.github.io/intergroup-contact-needs/Supplemental-Material-A-Annotated-Analysis>

For readers interested in the raw files, the raw RMarkdown file is available in our OSF repository (see Kreienkamp et al., 2022b) and can also be accessed as part of the full GitHub repository (Kreienkamp et al., 2022a).

# References

Kreienkamp, J., Agostini, M., Bringmann, L. F., de Jonge, P., & Epstude, K. (2022a). *intergroup-contact-needs [GitHub repositor: Materials, computer code]*. <https://janniscodes.github.io/intergroup-contact-needs/>

Kreienkamp, J., Agostini, M., Bringmann, L. F., de Jonge, P., & Epstude, K. (2022b). *Need Fulfillment During Intergroup Contact [OSF repository: Materials, data, code]*. <https://doi.org/10.17605/OSF.IO/PR9ZS>
