# Supplementary material for: Need Fulfillment During Intergroup Contact: Three Experience Sampling Studies
Source: Pers Soc Psychol Bull. 2023 Nov 21;51(6):1047–77. doi: 10.1177/01461672231204063 (PMC12044218; doi:10.1177/01461672231204063)
Supplement: sj-docx-3-psp-10.1177_01461672231204063 – Supplemental material for Need Fulfillment During Intergroup Contact: Three Experience Sampling Studies [file sj-docx-3-psp-10.1177_01461672231204063.docx]

Supplementary Information for

Need Fulfillment During Intergroup Contact: Three Experience

Sampling Studies

**Supplemental Information C: Motives Topic Model**

*Jannis Kreienkamp, Maximilian Agostini, Laura F. Bringmann, Peter de Jonge, Kai Epstude*

Corresponding Author: Jannis Kreienkamp

E-mail: j.kreienkamp@rug.nl

Last updated: June 1, 2023

1

# Supplemental Information C: Motives Topic Model

This supplementary information documents the methodology and results of the BERT topic model we conducted as part of embeddedness analyses. To make all supplemental materials as open as possible, we share the full Python Jupyter Notebook file which offers an annotated version of our all analysis steps. This file offers a transparent and reproducible analysis code, which includes full author commentary. An interactive HTML render of the notebook is part of our open GitHub repository. We recommend the rendered version for almost all readers (full Python code is also available via the rendered version).

<https://janniscodes.github.io/bert-migrant-need-content/Supplemental-Material-C-BERT-topic-model-outgroup>

For readers interested in the raw files, the raw Jupyter notebook is available in our OSF repository (see Kreienkamp et al., 2022b) and can also be accessed as part of the full GitHub repository (Kreienkamp et al., 2022a). Please note that this analysis used free text responses from our participants. To protect the privacy and confidentiality of our participants, we do not make the raw data openly accessible. If you would like to request access to the raw data, please reach out to the corresponding author.

# References

Kreienkamp, J., Agostini, M., Bringmann, L. F., de Jonge, P., & Epstude, K. (2022a). *intergroup-contact-needs [GitHub repositor: Materials, computer code]*. <https://janniscodes.github.io/intergroup-contact-needs/>

Kreienkamp, J., Agostini, M., Bringmann, L. F., de Jonge, P., & Epstude, K. (2022b). *Need Fulfillment During Intergroup Contact [OSF repository: Materials, data, code]*. <https://doi.org/10.17605/OSF.IO/PR9ZS>
