# Supplementary material for: Need Fulfillment During Intergroup Contact: Three Experience Sampling Studies
Source: Pers Soc Psychol Bull. 2023 Nov 21;51(6):1047–77. doi: 10.1177/01461672231204063 (PMC12044218; doi:10.1177/01461672231204063)
Supplement: sj-docx-4-psp-10.1177_01461672231204063 – Supplemental material for Need Fulfillment During Intergroup Contact: Three Experience Sampling Studies [file sj-docx-4-psp-10.1177_01461672231204063.docx]

Supplementary Information for

Need Fulfillment During Intergroup Contact: Three Experience

Sampling Studies

**Supplemental Information D: Coding Protocol Goal Directedness**

*Jannis Kreienkamp, Maximilian Agostini, Laura F. Bringmann, Peter de Jonge, Kai Epstude*

Corresponding Author: Jannis Kreienkamp

E-mail: j.kreienkamp@rug.nl

Last updated: June 1, 2023

1

# Supplemental Information D: Coding Protocol Goal Directedness

As part of our robustness analyses, we developed a coding protocol to assess the goal-directedness of participants’ free-text responses regarding their main goal during interactions with outgroup members. The coding captures two dimensions: practical needs and underlying psychological needs. Two independent coders coded each response, and the dimensions were assessed separately to ensure the validity and reliability of the coding process. The full coding protocol of available as part of our OSF repository (see Kreienkamp et al., 2022):

<https://osf.io/tye9s?view_only=88f136ec6704423b84c6ea1998d780a1>

# References

Kreienkamp, J., Agostini, M., Bringmann, L. F., de Jonge, P., & Epstude, K. (2022). *Need Fulfillment During Intergroup Contact [OSF repository: Materials, data, code]*. <https://doi.org/10.17605/OSF.IO/PR9ZS>
